# Multidrug resistance and inappropriate empiric therapy as predictors of hospital stay in diabetic foot infections

**DOI:** 10.1371/journal.pone.0350837

**Published:** 2026-06-08

**Authors:** Lana Zuriegat, Rania Itani, Khawla Abu Hammour, Rana Abu-Farha

**Affiliations:** 1 Clinical Pharmacy and Therapeutics Department, Faculty of Pharmacy, Applied Science Private University, Amman, Jordan; 2 Pharmacy Practice Department, Faculty of Pharmacy, Beirut Arab University, Beirut, Lebanon; 3 Department Biopharmaceutics and Clinical Pharmacy, Faculty of Pharmacy, The University of Jordan, Amman, Jordan; Institute of Medicine (IOM), Maharajgunj Medical Campus, TU, NEPAL

## Abstract

**Objectives:**

This study explored the causative pathogens, resistance patterns, and treatment appropriateness for diabetic foot infections (DFI) at a tertiary care center in Jordan.

**Methods:**

A retrospective review was conducted on 234 patients diagnosed with DFIs at a tertiary care center in Jordan. Data collected included patient demographics, diabetes history, infection severity, culture results, and antibiotic treatment details. Bacterial isolates were classified as multidrug-resistant (MDR) or non-MDR based on standard definitions. Therapy was considered appropriate if at least one antibiotic given within the first 48 hours of admission covered all identified pathogens and was administered with the correct dosage, formulation, and route. Statistical analyses examined the relationship between resistance patterns, treatment adequacy, and hospital stay duration.

**Results:**

The most frequently isolated bacterium was *Staphylococcus aureus*, identified in 117 cases (50.0%), including MRSA (n = 46, 19.6%) and MSSA (n = 71, 30.3%). This was followed by *Escherichia coli* in 33 cases (14.1%) and *Pseudomonas aeruginosa* in 32 cases (13.7%). MDR organisms accounted for 152 infections (65%). Empiric therapy was deemed appropriate in 111 patients (47.4%), inappropriate in 74 (31.6%), and not assessable due to missing data in 49 (20.9%). Following culture and susceptibility results, antibiotics in 87 cases (37.2%) remained unchanged. In multivariate analysis, only infection severity was significantly associated with prolonged hospitalization (β = −0.161, *P* = 0.034).

**Conclusion:**

MDR organisms were common in DFIs but not significantly associated with prolonged hospitalization. Infection severity was the key predictor of length of stay. These findings highlight the importance of early infection assessment, appropriate empirical therapy based on local resistance patterns, and robust antibiotic stewardship.

## 1. Background

In 2022, an estimated 828 million adults were living with diabetes mellitus—a striking increase of over 630 million cases since 1990 [1]. This growing burden poses significant challenges to healthcare systems and drives substantial increases in healthcare costs globally [2].

A poorly regulated diabetes mellitus generally gives rise to multiple major complications associated with diabetic foot infections (DFI’s). The identification and classification of the severity of the infection is an important segment of providing efficient care to the patient. The Infectious Disease Society of America (IDSA) and The International Working Group on the Diabetic Foot (IWGDF) have established grading criteria that allow for the classification of DFI’s from mild to moderate to severe, using the clinical manifestations of DFI’s: such as the amount of inflammation, how far down the tissue the infection has spread, and whether or not any bone is infected [3,4]. Severe infections often result in extended hospitalization and aggressive treatment of the infection [5].

Amid increasing antimicrobial resistance, prompt initiation of appropriate empirical antibiotic therapy is essential. The IDSA guidelines recommend broad-spectrum antibiotics targeting the typical pathogens found in DFIs—including gram-positive, gram-negative, and anaerobic bacteria—while awaiting culture results [6]. Subsequent treatment adjustments should be based on microbiological findings and susceptibility profiles.

Many studies have been conducted in Jordan examining elements of DFIs such as the prevalence of pathogens, the pattern of resistance among these pathogens, or virulence factors exhibited by particular organisms [7–9]. However, there are currently no studies conducted in Jordan that have evaluated the appropriateness of empirically treating DFIs with antimicrobial agents or the relationship between the empirical treatment used and the clinical outcome of length of stay. The effect of MDR infections on the length of stay in Jordan also remains unstudied. Therefore, the objective of this study was to describe the microbial spectrum associated with DFIs as well as the associated resistance patterns. In addition, we assessed the appropriateness of empirically prescribed antibiotic therapy and analyzed the association between the empirically prescribed therapy and subsequent clinical outcomes, specifically length of stay.

## 2. Methods

### 2.1. Study design and setting

A retrospective observational research design was conducted to recruit patients 18 years old and older who had been admitted to the hospital with DFIs as subjects. The research was conducted at Jordan University Hospital (JUH), an academic medical facility in Amman Jordan that is established as of 1971 and is a 600-bed institution offering 64 different types of specialized care. Patients with DFIs during the time frame from January 1, 2015 – September 1,2024 were located by a computer-assisted review of the electronic healthcare records system for the purposes of this study. As this was a retrospective observational study, no a priori sample size calculation or power analysis was performed. Instead, the sample size was determined by the number of eligible cases that met the inclusion criteria during the defined study period. Data were accessed for research purposes, with data collection initiated on October 1, 2024.

### 2.2. Inclusion and exclusion criteria

The initial list of patients was obtained from the Information Technology Department at JUH and included all individuals admitted with DFIs during the study period identified using relevant ICD-10 codes. Patients were eligible for inclusion if they were 18 years or older, admitted to either the internal medicine or surgical departments, diagnosed with a diabetic foot infection (regardless of diabetes type), and received at least one antibiotic during hospitalization. Patients were excluded if they lacked susceptibility testing results, had no available or negative culture results, were managed as outpatients, or had concurrent infections. Additional exclusions applied to pregnant or lactating women and individuals with foot infections resulting from other chronic conditions, such as tuberculosis, malignancy, trauma, non-diabetic neuropathy, or arterial and venous disorders.

### 2.3. Data collection form

The data collection instrument was constructed following extensive literature review [10,11]. It had seven separate domains. The first domain consisted of demographics and admission-related data about the patient such as date of birth, sex, smoking history, date of admission, date of discharge, source of admission (e.g., emergency department versus outpatient), and whether the patient was transferred to intensive care unit (ICU). The second domain gathered diabetic related medical data such as type and duration of Diabetes, currently ordered Anti-diabetic medications, and associated complications (e.g., diabetic foot ulcer (DFU), gangrene, amputation history, and/or osteomyelitis). Comorbid medical conditions and most recent Glycosylated Hemoglobin (HbA1c) results were also documented. The third domain determined the severity of infection using the IWGDF/IDSA Infectious Disease classification system when available [12]. The fourth domain focused on details of specimen collection related to the date(s) and types of specimens submitted for culture. The fifth domain detailed empirical antibiotic therapy, which included indications for choice of drug therapy, how the drug was administered, dosages prescribed and when drug therapy was started, and the duration of treatment. The sixth domain collected data on antibiotic susceptibility and the resistance and classification patterns of the isolates. Finally, the seventh domain detailed definitive antibiotic therapy and whether any changes were made.

### 2.4. Study outcomes

Our study looked at three main outcomes. First, we examined the resistance patterns of bacterial isolates from DFI cases, classifying them as either non-multidrug-resistant (non-MDR) or multidrug-resistant (MDR) based on antimicrobial susceptibility testing. Antimicrobial susceptibility testing was conducted using the disc diffusion method in accordance with the Clinical and Laboratory Standards Institute (CLSI) recommendations [13]. Non-MDR bacteria were sensitive to all tested antimicrobials or resistant to only one or two classes. MDR bacteria were defined as non-susceptible to at least one agent in three or more antimicrobial categories, according to the standardized international definition proposed by Magiorakos et al. (2012) [14]. For infections with multiple bacteria, if any isolate met the MDR criteria, the whole infection was classified as MDR.

The second outcome focused on how appropriate the empiric antibiotic therapy was (**Table 1**). Appropriate treatment was defined as receiving at least one antimicrobial for which the identified pathogen was found to be susceptible *in vitro*, and was administered with the correct dosage, formulation, and route [15]. In polymicrobial infection, all identified pathogens needed to be covered by at least one antimicrobial drug to be classified as appropriate therapy [15]. In our study we assessed the appropriateness within the 48 hours of hospital admission. If any of these factors were lacking, the therapy was deemed inappropriate [15]. Cases where susceptibility data was missing or incomplete were labeled as having insufficient information, making it impossible to determine the appropriateness of the empiric treatment. Specifically, this included cases lacking verifiable administration timestamps or those where susceptibility testing was not performed for all isolated pathogens.

**Table 1:** Operational definitions for empirical antibiotic appropriateness.

Category	Criteria for “Appropriate” Classification
Microbiological Concordance	All isolated pathogens must be susceptible *in vitro* to at least one empirical agent. In polymicrobial infections, 100% of isolates must be covered [15].
Dosing & Route	Dose, formulation, and route must align with IWGDF/IDSA 2024 standards, adjusted for admission renal function [12].
Timing	First dose must be administered within 48 hours of hospital admission.
Insufficient Data	Assigned when susceptibility results were incomplete for any isolate or when medication administration timestamps were missing from the electronic record.

The third outcome examined in this study was the duration of hospitalization. Specifically, we assessed how factors such as antimicrobial resistance patterns and the appropriateness of initial antibiotic therapy influenced the length of stay. For this analysis, length of stay was calculated as the total number of days from the patient’s admission to discharge, encompassing the entire hospital stay regardless of whether each day was directly related to infection treatment. In addition to antimicrobial factors, we considered a range of demographic and clinical variables as potential contributors to prolonged hospitalization. These included age, gender, smoking status, type of diabetes, HbA1c levels, department of admission (internal medicine vs. surgical wards), ICU transfer during hospitalization, presence of diabetic foot ulcers, and infection severity based on the IWGDF/IDSA classification.

### 2.5. Ethical considerations

This study was conducted in accordance with the ethical principles of the World Medical Association’s Declaration of Helsinki. The research protocol was approved by the Institutional Review Board (IRB) at Jordan University Hospital (approval code: 10/2024/23195). Data collection involved a comprehensive review of medical records containing identifiable information on individual participants. However, the research team did not contact or follow up with patients. Given the retrospective and observational nature of the study, which utilized post-discharge data while ensuring participant confidentiality, the IRB of the participating hospital granted a waiver of informed consent.

### 2.6. Handling of missing data

No imputation was performed for missing data. Patients with incomplete or unavailable culture and susceptibility results were excluded from analyses evaluating the appropriateness of empiric antibiotic therapy. These patients were retained in other analyses when relevant data were available.

### 2.7. Statistical analysis

All statistical analyses were conducted using IBM SPSS^®^ version 26. Descriptive statistics were used to summarize the data. Categorical variables were reported as frequencies and percentages, while continuous variables were expressed as means with standard deviations (SD) for normally distributed data, or as medians with interquartile ranges (IQR) for non-normally distributed data. The Kolmogorov–Smirnov test was used to assess the normality of continuous variables, with a p-value greater than 0.05 indicating a normal distribution.

To identify independent predictors of hospital length of stay, linear regression analysis was conducted in two steps. First, univariate linear regression was performed for each predictor variable using log-transformed LOS as the outcome. Variables with a p < 0.25 in the univariate regression were considered for inclusion in the multivariate linear regression model. The multivariate regression model was built using the enter method. Multicollinearity was evaluated using the variance inflation factor (VIF), with values above 5 indicating potential multicollinearity. A p < 0.05 was considered statistically significant in all analyses.

## 3. Results

### 3.1. Screened and enrolled cases

A total of 1,145 DFI cases were screened for inclusion. Of these, 234 cases met the eligibility criteria and were included in the analysis. The remaining 911 cases were excluded for the following reasons: 545 lacked adequate susceptibility or culture data, 119 had negative culture results, 140 were managed as outpatients, and 107 had concurrent infections.

### 3.2. Demographic and medical characteristics

Among included patients, 177 (75.6%) were male and 57 (24.4%) female. Most were aged 55 or older (n = 189, 80.8%). The majority were admitted through the emergency department (n = 181, 77.4%), and to the internal medicine department (n = 169, 72.2%), while 27.8% (n = 65) were admitted to surgery.

Chronic medical conditions were recorded in 182 patients (77.8%), mainly hypertension (n = 164, 70.1%) and cardiovascular disease (n = 101, 43.2%). ICU transfers occurred in 36 patients (15.4%). The median hospital stay was 12 days (IQR = 12). Full patient characteristics are detailed in **Table 2**.

**Table 2:** Demographic and medical characteristics of the study participants (n = 234).

Parameter	Median (IQR)	Frequency (%)
Gender		
Male		177 (75.6)
Female		57 (24.4)
Age categories		
Less than 35		2 (0.9)
35-44		7 (3.0)
45-54		36 (15.4)
55-64		81 (34.6)
Equal or more than 65		108 (46.2)
Smoking status		
Non-smoker		101 (43.2)
Ex-smoker		24 (10.3)
Smoker		46 (19.7)
Missing value		63 (26.9
Department of admission		
Internal medicine		169 (72.2)
Surgery		65 (27.8)
Admission source		
Outpatient clinic		53 (22.6)
Emergency department		181 (77.4)
Length of hospital stay (days)	12.0 (12.0)	
Health status		
Healthy		52 (22.2)
With chronic condition		182 (77.8)
Most common chronic illnesses		
Hypertension		164 (70.1)
Cardiovascular disease		101 (43.2)
Chronic kidney disease		52 (22.2)
Was the patient transferred to the ICU		
Yes		36 (15.4)
No		198 (84.6)

IQR: Interquartile range.

### 3.3. Diabetes-related medical information

Diabetes-specific data are shown in **Table 3**. Most patients had type 2 diabetes (n = 230, 98.3%), while type 1 was rare (n = 4, 1.7%). HbA1c indicated uncontrolled glycemia in 148 patients (63.2%), and controlled in 37 patients (15.8%).

**Table 3:** Diabetes-related medical information and treatment regimens of participants (n = 234).

Parameter	Frequency (%)
Type of diabetes	
Type 1	4 (1.7)
Type 2	230 (98.3)
Type of antidiabetics prescribed	
Insulin	194 (82.9)
Metformin	66 (28.2)
Sulfonylureas	23 (9.8)
Thiazolidinediones	9 (93.8)
Dipeptidyl peptidase 4 (DPP-4) inhibitors	1 (0.4)
HBA1C value	
Controlled	37 (15.8)
Uncontrolled	148 (63.2)
Missing	49 (20.9)
Number of prescribed antidiabetics per patient	
One	181 (77.4)
Two	48 (20.5)
Three	4 (1.7)
Four	1 (0.4)
Diabetic related complications	
Neuropathy	13 (5.6)
Retinopathy	9 (3.8)
Nephropathy	52 (22.2)
Peripheral vascular complications	13 (5.6)
Diabetic foot related conditions	
Diabetic foot ulcer	229 (97.9)
History of amputation	75 (32.1)
History of gangrene	19 (8.1)
IWGDF/IDSA classification of DFI	
Mild (Grade 2)	20 (8.5)
Moderate (Grade 3)	135 (57.7)
Severe (Grade 4)	30 (12.8)
Missing data	49 (20.9)

# Controlled HbA1c is defined as an HbA1c level of less than 7% for all patients, except for elderly patients or those at risk of hypoglycemia, for whom controlled HbA1c is less than 8%. IWGDF/IDSA: International Working Group on the Diabetic Foot/Infectious Diseases Society of America.

Monotherapy was prescribed for 77.4% of the patients (n = 181). Insulin was the most used (n = 194, 82.9%), followed by metformin (n = 66, 28.2%), sulfonylureas (n = 23, 9.8%), thiazolidinediones (n = 9, 3.8%), and DPP-4 inhibitors (n = 1, 0.4%).

Common complications included nephropathy (n = 52, 22.2%), neuropathy (n = 13, 5.6%), peripheral vascular complications (n = 13, 5.6%), and retinopathy (n = 9, 3.8%). DFUs were seen in 229 patients (97.9%). Prior amputation and gangrene were reported in 75 patients (32.1%) and 19 patients (8.1%), respectively. Severity of infection (IWGDF/IDSA) was mild in 20 patients (8.5%), moderate in 135 patients (57.7%), and severe 30 patients (12.8%).

### 3.4. Isolated bacteria

Skin swab cultures were the most frequently collected specimen type (n = 185, 79.1%), followed by blood cultures (n = 49, 20.9%). Most patients had one bacterial isolate (n = 135, 57.7%), while 79 (33.8%) had two isolates. A smaller number had three (n = 16, 6.8%) or four (n = 4, 1.7%) isolates.

*Staphylococcus* species were the most commonly isolated organisms (n = 117, 50%), including methicillin-resistant *Staphylococcus aureus* (MRSA) in 46 cases (19.6%). *Enterococcus* species were identified in 51 cases (21.8%). Other frequent pathogens included *Escherichia coli* (n = 33, 14.1%) and *Pseudomonas aeruginosa* (n = 32, 13.7%). Other organisms were less commonly detected.

### 3.5. Empirical and definitive antimicrobial therapy

All patients received empiric antimicrobial therapy (n = 234, 100%). Two antibiotics were prescribed in 89 cases (38.0%), one antibiotic in 85 cases (36.3%), three antibiotics in 55 cases (23.5%), and four or more in 5 cases (2.1%). Most antibiotics were administered intravenously (n = 179, 76.5%), while 31 patients (13.2%) received oral antibiotics.

**Fig 1:**
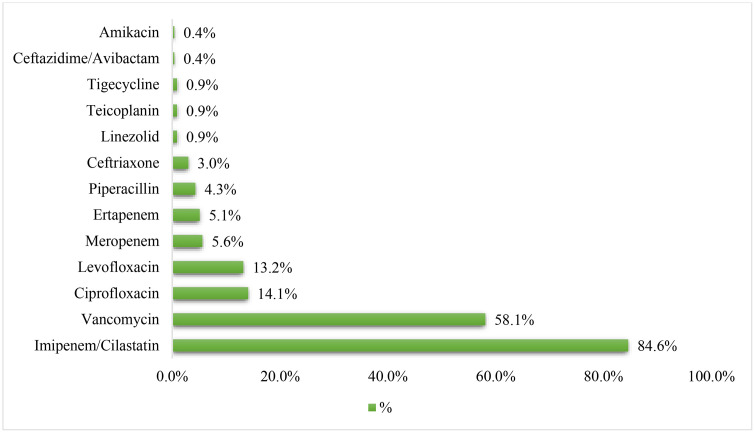
Frequency of empiric antibiotics prescribed to study participants (n = 234).

Imipenem-cilastatin was the most commonly prescribed empiric agent (n = 198, 84.6%), followed by vancomycin (n = 136, 58.1%). Other empiric antibiotics included ciprofloxacin (n = 33, 14.1%), levofloxacin (n = 31, 13.2%), and meropenem (n = 13, 5.6%). These patterns are illustrated in **Fig 1**.

Following culture and susceptibility results, antibiotic therapy was modified in several ways (**Table 4**). In 87 cases (37.2%), therapy remained unchanged. In 34 patients (14.5%), antibiotics were switched, and in 29 (12.4%), additional antibiotics were added. Antibiotics were discontinued in 20 cases (8.5%) based on susceptibility findings. Some patients were discharged (n = 63, 26.9%) or died (n = 1, 0.4%) before culture results were available.

**Table 4:** Clinical management and modification of antibiotic therapy (N = 234).

Clinical Status and Subsequent Action	n (%)
**I. Remained Hospitalized (Modification Opportunity)**	**170 (72.7)**
Continued empirical regimen (Unchanged)	87 (37.2)
Antimicrobial switch	34 (14.5)
Addition of supplemental antibiotic	29 (12.4)
Discontinuation of antibiotic therapy	20 (8.5)
**II. Discharged or Deceased Prior to Culture Results**	**64 (27.3)**
Patient discharged	63 (26.9)
In-hospital mortality	1 (0.4)
**Total**	**234 (100)**

Regarding definitive therapy, 73 patients (31.2%) received two antibiotics, 64 (27.4%) received none, 51 (21.8%) received one, 29 (12.4%) received three, and 17 (7.3%) received four or more. The top five definitive antibiotics were imipenem-cilastatin (n = 133/355, 37.5%), vancomycin (n = 105/355, 29.6%), ciprofloxacin and levofloxacin (each n = 30/355, 8.5%), and piperacillin-tazobactam (n = 12/355, 3.4%).

### 3.6. Antimicrobial resistance patterns

Multidrug-resistant (MDR) bacteria were identified in 152 patients (65%), while 82 patients (35%) had infections caused by non-MDR organisms.

### 3.7. Appropriateness of antimicrobial therapy

**Fig 2:**
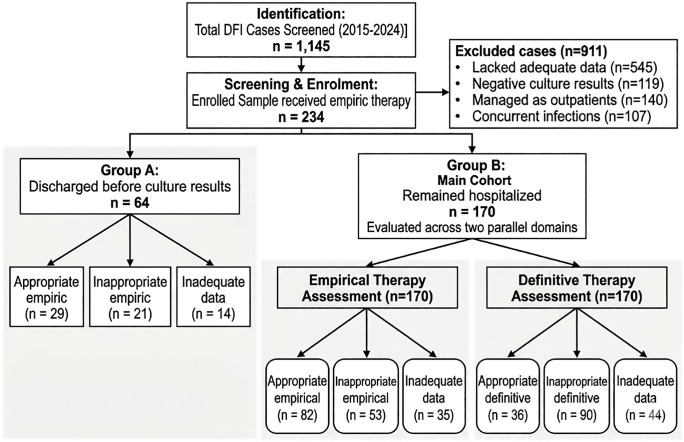
Flow diagram of the study population identification, inclusion, and analysis.

As shown in **Fig 2**, empiric therapy was deemed appropriate in 111 patients (47.4%), inappropriate in 74 (31.6%), and not assessable due to missing data in 49 (20.9%). Among 170 patients who received definitive therapy, 36 (21.2%) received appropriate therapy, 90 (52.9%) received inappropriate therapy, and 44 (25.9%) lacked sufficient data for evaluation.

### 3.8. Predictors of length of hospital stay

A multivariate linear regression analysis was conducted to identify factors associated with the log-transformed length of hospital stay among patients with DFIs (**Table 5**). In the univariate analysis, seven predictors had *P* values less than 0.250, making them eligible for inclusion in the multivariate linear regression model. These included type of diabetes (*P* = 0.105), department of admission (*P* = 0.048), ICU transfer (*P* = 0.011), presence of diabetic foot ulcer (*P* = 0.051), infection severity based on the IWGDF/IDSA classification (*P* = 0.015), appropriate empiric therapy (*P* = 0.234), and presence of MDR infection (*P* = 0.069).

**Table 5:** Predictors of length of hospital stay in patients with diabetic foot infections: a multivariate log-transformed linear regression model.

Predictors	Dependent variable: Log-Transformed Length of Stay			
	Beta	P-value#	Beta	P-value$
Age (years)	−0.018	0.784	---	---
Gender				
Male	Reference			
Female	−0.057	0.385	---	---
Smoking Status				
Smoker	Reference			
Non-smoker/Exsmoker	0.001	0.982	---	---
Type of Diabetes				
Type 1	Reference			
Type 2	−0.106	0.105^	0.095	0.197
HbA1c (%)	0.043	0.564	---	---
Department of admission				
Internal Medicine	Reference			
Surgery	0.129	0.048^	−0.005	0.946
ICU Transfer				
Yes	Reference			
No	−0.167	0.011^	−0.097	0.200
Diabetic Foot Ulcer Present				
Yes	Reference			
No	−0.128	0.051^	−0.101	0.178
Severity of infection (IWGDF/IDSA)				
Grade 3 or 4	Reference			
Grade 2	−0.178	0.015^	−0.161	0.034*
Appropriate empiric therapy				
Yes	Reference			
No/Insufficient	0.078	0.234^	0.093	0.227
MDR infection				
Yes	Reference			
No	0.119	0.069^	0.079	0.286

# Using univariate linear regression, $ Using multivariate linear regression, ^ Eligible for entry in multivariate linear regression, * Significant at 0.05 significance level.

After adjusting for covariates in the multivariate model, only the severity of infection remained a statistically significant predictor. Patients with less severe infections (IWGDF/IDSA Grader 2) had significantly shorter hospital stays compared to those with more severe infections (Grade 3 or 4; β = −0.161, P = 0.034). The multivariate linear regression model showed a weak explanatory power for length of hospital stay (R² = 0.071, adjusted R² = 0.034).

## 4. Discussion

This study explored the epidemiology, causative pathogens, resistance patterns, and treatment approaches for DFI at a tertiary care center in Jordan. A total of 234 patients participated, with males representing 75.6% (n = 177) and females 24.4% (n = 57).The most commonly isolated pathogens were *Staphylococcus aureus* (including MRSA), *Enterococcus* species, *Escherichia coli*, *Pseudomonas aeruginosa*, *Proteus mirabilis*, and *Klebsiella pneumoniae*. These findings are consistent with global reports identifying *Staphylococcus aureus* as a leading cause of DFIs, along with a rising incidence of *Pseudomonas aeruginosa* [4]. Similar pathogen profiles were reported by Dörr et al. and Son et al., who highlighted predominance of Gram-positive bacteria like *Staphylococcus aureus* and *Enterococcus faecalis*, as well as significant presence of Gram-negative species [16,17]. While *Staphylococcus aureus* remains a leading pathogen globally, the notable presence of Gram-negative bacteria such as *Pseudomonas aeruginosa* highlights the importance of considering local microbiological patterns that may differ due to regional factors such as environmental conditions and healthcare practices [18]. These results support the need for empirical antibiotic therapy that covers both Gram-positive and Gram-negative bacteria, including MDR strains such as MRSA and *Pseudomonas aeruginosa*.

Antimicrobial resistance is still a major issue when treating DFIs. In this study, the authors found that 65% of all infections were from MDR bacteria with *Staphylococcus aureus* being the most common. Other studies in different places have shown that the prevalence of MDR bacteria ranges from 48% to 80% [19–21]. The presence of MDR pathogens makes it harder to select appropriate treatments and can result in longer lengths of stay in hospitals and more cost to the healthcare system [21,22]. These results highlight the need for strong and effective antibiotic stewardship programs as a way to help limit the emergence of resistance.

Appropriate antibiotic selection should be based upon local antibiograms, the risk factors/protocols for MDR development for each patient and the severity of the infection [4]. As the prevalence of MDR infections increases, the use of broad-spectrum empirical antibiotics such as imipenem-cilastatin (84.6%) and vancomycin (58.1%) has increased dramatically. This has been shown to be consistent with the International Working Group on the Diabetic Foot (IWGDF)/Infectious Disease Society of America (IDSA) guidelines that advocate broad-spectrum coverage in patients with severe DFIs or suspected to have had an MDR pathogen [12]. Broadly defined empiric antibiotic coverage is essential to providing proper management of DFIs [23].

Despite this, nearly one-third (31.6%) of patients received inappropriate empirical antibiotic therapy. Reasons for this may include limited access to timely culture and sensitivity results, lack of updated local antibiograms, and variability in clinical decision-making, especially in the absence of standardized local protocols [24]. This finding echoes results from Aviatin et al., where 46% of cases involved suboptimal initial treatment [25]. Enhancing adherence to international guidelines and developing national protocols can help clinicians optimize antibiotic choice, improve outcomes, reduce hospital stays, and lower healthcare expenditures [26,27].

Regarding antibiotic adjustments after culture results, over one-third of patients (37.2%) continued on broad-spectrum antibiotics rather than switching to narrower-spectrum agents as recommended. This reflects a potential gap in antimicrobial de-escalation. This phenomenon, commonly described as “clinical inertia,” suggests that clinicians may perceive ongoing success of an initial broad-spectrum regimen as safer than changing to a narrow-spectrum regimen; however, this perception is contrary to culture results. It is likely that the underutilization of available antibiogram data has been further repeated by the lack of dedicated multidisciplinary antimicrobial stewardship teams and formalized antimicrobial time-out protocols in many hospitals throughout Jordan. Without requiring a therapy end review of each patient at 48–72 hours, use of broad-spectrum antibiotics will remain, thus increasing the likelihood of future collateral damage and the risk of developing resistance..

In this study, neither the appropriateness of empirical antibiotic therapy nor the presence of MDR infections showed a statistically significant association with log transformed length of hospital stay. Although global reports have reported a significant association between MDR and extended length of stay, our results suggest a more complex relationship between these variables. The high prevalence of MDR organisms in a Jordanian tertiary care center may counteract by the early and aggressive use (i.e., “neutralizing) of broad-spectrum empirically administered agents (i.e., carbapenems and vancomycin), which effectively “neutralized” the resistance risk before it could translate into clinical failure.

Importantly, this study identified infection severity, as classified by the IWGDF/IDSA system, as the only independent predictor of prolonged hospitalization. Hospital stays were much longer for patients diagnosed with moderate to severe grade (3 and 4) infection compared to those who were rated as having mild infection [2]. Previous studies have found that higher levels of infection severity correlate to longer lengths of stay [28,29]. Wukich et al. found patients who had severe DFIs had longer lengths of stay in the hospital than patients with milder types of diabetic foot infection [28], and Choi et al.‘s retrospective study also found a significant association between severity of the wounds and longer lengths of stay in patients with diabetic foot after adjusting for other clinical factors [29]. These studies underscore how important it is to recognize moderate to severe infections and provide early aggressive therapy to potentially save time in the hospital and improve outcomes.

This research has certain limitation, including the limited ability to generalize because of the single-center design, which has different regional approaches to treatment and resistance patterns. The lack of long-term follow-up restricts knowledge about the outcomes from being hospitalized. The number of people included in the sample limited the statistical power of the subgroup analyses, especially considering the prevalence of these infections. A post hoc analysis shows the overall regression model has limited explanatory power (R2 = 0.071) but still had acceptable overall statistical power. However, subgroup analyses are still probably underpowered. Consequently, caution must be taken when interpreting these results. Additionally, electronic medical records have potential data gaps and errors that further hinder using retrospective cohorts. Moreover, the predominantly incomplete microbiological data used for selecting cases out of those that were screened for this study may be subject to selection bias, which has the potential to inflate the representation of more complicated or worse infections than what was observed for the overall pathogen distribution and resistance rates, and that were also likely to be more complicated in nature. Moreover, the retrospective design of this study limited the ability to evaluate the clinical decision-making processes that were associated with selecting the initial empirical therapy and the eventual changes to the original empirical therapy.

## 5. Conclusion

This study explored the causative pathogens, antimicrobial resistance patterns, and treatment approaches for DFIs at a tertiary care center in Jordan. The results show that the most common pathogens involved are Gram-positive bacteria, especially *Staphylococcus aureus*, along with Gram-negative bacteria such as *Pseudomonas aeruginosa*, *Escherichia coli*, and *Klebsiella pneumoniae*. While MDR organisms were frequently encountered, their presence was not significantly associated with prolonged hospital stay. Instead, infection severity, as classified by the IWGDF/IDSA system, was the key factor linked to longer hospitalization. These findings emphasize the importance of using antibiotic therapies tailored to local resistance patterns and highlight the critical role of robust antibiotic stewardship programs in improving patient care and treatment effectiveness.

## Supporting information

S1 Table Detailed antimicrobial susceptibility profiles by organism. (XLSX)
