# Supplementary material for: Multidrug resistance and inappropriate empiric therapy as predictors of hospital stay in diabetic foot infections
Source: PLoS One. 2026 Jun 8;21(6):e0350837. doi: 10.1371/journal.pone.0350837 (PMC13245766; doi:10.1371/journal.pone.0350837)
Supplement: Attachment — Submitted filename: Comments PloS One.docx [file pone.0350837.s002.docx]

**Reviewers’ Report**

**Title: Multidrug Resistance and Inappropriate Empiric Therapy as Predictors of Hospital Stay in Diabetic Foot Infections**

**General Comments**

The manuscript addresses an important and clinically relevant question. However, it has substantial methodological and reporting deficiencies that compromise data integrity, clarity, and policy compliance. Key problems include inconsistencies in reported results, unclear definitions, insufficient statistical rigor, and inadequate discussion of limitations.

I recommend major revision (bordering on rejection unless the authors can perform substantial re-analysis and fully reconcile inconsistencies). Without these changes, the study’s validity and interpretability remain doubtful.

**Major Issues**

***Core Data Inconsistency***

Multiple discrepancies between Abstract and Results (e.g., S. aureus in Abstract: 88 cases [37.6%] vs. Staphylococcus spp. in Results: 117 [50%]). LOS reported as mean in Abstract vs. median in Results. These inconsistencies occur throughout the paper. All counts, percentages, and summary measures must be reconciled and harmonized.

***Missing Data Reporting***

Abstract omits the number of patients excluded due to missing data (20.9%). This must be explicitly stated.

***Lack of Research Gap Justification***

The manuscript does not clearly explain the novelty of the study in the Jordanian context or how it adds insight beyond existing literature.

***Empiric Therapy Assessment***

It is unclear whether both antimicrobial spectrum coverage and dosing adequacy were systematically evaluated.

***Handling of Missing Data***

No description is provided for how missing data (20.9% for appropriateness assessment) were addressed statistically.

***Length of Stay (LOS) Definition***

It is unclear whether LOS is counted from admission to discharge or only for infection-related days.

***No Power/Sample Size Calculation***

The study lacks any sample size justification or power analysis.

***Retrospective Design Limitations***

Methodological limitations inherent to retrospective design are not discussed. Operational definitions, missing data handling, and rationale for statistical tests must be clarified.

***Results Presentation***

Results are repetitive, descriptive data in text duplicate tables. Resistance analysis is superficial and no organism-specific resistance rates are presented. The counts across Abstract, Results, and Tables do not match. Include a supplementary table with detailed susceptibility profiles per organism and consistent denominators.

Shapiro–Wilk test is acceptable, but for sample sizes ≥50, tests like Kolmogorov–Smirnov are often preferred. Rationale for choice should be stated. LOS is influenced by severity, comorbidities, ICU stay, etc. Simple two-group comparisons risk bias. Authors should clarify whether these factors were considered. A multivariate linear regression (with log-transformed LOS) or Cox proportional hazards model (for discharge time) would allow adjustment for confounders. I recommend re-running the statistical analysis accordingly.

***Discussion Interpretation***

Overstates similarity to other studies without considering local microbiology patterns. Does not explore possible causes for high inappropriate empiric therapy rates. LOS is significant for therapy appropriateness but not MDR status and the reasons are not discussed.

***Integration with Local Context***

Discussion should link observed patterns to local clinical practices (e.g., empiric prescribing habits, lab turnaround times).
