# Supplementary material for: Multidrug resistance and inappropriate empiric therapy as predictors of hospital stay in diabetic foot infections
Source: PLoS One. 2026 Jun 8;21(6):e0350837. doi: 10.1371/journal.pone.0350837 (PMC13245766; doi:10.1371/journal.pone.0350837)
Supplement: Attachment — Submitted filename: Recommendation_AY.docx [file pone.0350837.s003.docx]

**Overall Recommendation:** Major Revisions

**Overall Impression:**
This manuscript presents a valuable retrospective analysis of diabetic foot infections (DFIs) at a tertiary care center in Jordan. The manuscript is generally well-structured. However, major concerns regarding potential selection bias, data analysis, and the interpretation of results must be addressed before the manuscript can be considered for publication.

**Detailed Comments:**

1. The exclusion of 79.6% of screened cases (911/1145) of screened cases creates significant selection bias, likely over-representing complex infections. Therefore, the pathogen prevalence and MDR rates cannot be considered representative of the center's true DFI population and must be highlighted as a major limitation.
2. The claim that inappropriate empiric therapy independently prolongs hospital stay is not supported by the current analysis. The analysis must account for confounding by infection severity and other clinical factors. A multivariate model is essential to establish this relationship.
3. There are discrepancies between the Abstract and the main Results section (3.4) that must be reconciled for the manuscript to be accurate and credible.
   a. *Abstract:* S. aureus = 88 (37.6%); *Results:* Staphylococcus species = 117 (50%). Please clarify the breakdown between S. aureus (MSSA+MRSA) and other Staphylococci.
   b. *Abstract:* P. aeruginosa = 40 (17.1%); *Results:* P. aeruginosa = 32 (13.7%).
   c. *Abstract:* E. coli = 35 (15.0%); *Results:* E. coli = 33 (14.1%).
      Please carefully review and ensure all data points are consistent throughout the
4. The flowchart (Figure 2) is currently confusing and difficult to follow. The numbers in the boxes (e.g., "29 received appropriate...") do not match the totals in the text. This figure should be redesigned for clarity to accurately visually represent the patient flow from screening through the assessment of empiric and definitive therapy.
5. Please define "concurrent infections" in the exclusion criteria. What types of infections were excluded (e.g., pneumonia, UTI)?
