# Supplementary material for: Multidrug resistance and inappropriate empiric therapy as predictors of hospital stay in diabetic foot infections
Source: PLoS One. 2026 Jun 8;21(6):e0350837. doi: 10.1371/journal.pone.0350837 (PMC13245766; doi:10.1371/journal.pone.0350837)
Supplement: Attachment — Submitted filename: Reply to reviewers.docx [file pone.0350837.s006.docx]

Reviewer #1:

General Comments

The manuscript addresses an important and clinically relevant question. However, it has substantial methodological and reporting deficiencies that compromise data integrity, clarity, and policy compliance. Key problems include inconsistencies in reported results, unclear definitions, insufficient statistical rigor, and inadequate discussion of limitations.

I recommend major revision (bordering on rejection unless the authors can perform substantial re-analysis and fully reconcile inconsistencies). Without these changes, the study’s validity and interpretability remain doubtful.

Major Issues

Core Data Inconsistency

Multiple discrepancies between Abstract and Results (e.g., S. aureus in Abstract: 88 cases [37.6%] vs. Staphylococcus spp. in Results: 117 [50%]). LOS reported as mean in Abstract vs. median in Results. These inconsistencies occur throughout the paper. All counts, percentages, and summary measures must be reconciled and harmonized.

Response

Thank you for pointing out the discrepancies between the Abstract and the Results section. These inconsistencies were inadvertently introduced. We have carefully reviewed and corrected all relevant data in the result section of the abstract as follows:

“Results: The most frequently isolated bacterium was *Staphylococcus aureus*, identified in 117 cases (50.0%), including MRSA (n = 46, 19.6%) and MSSA (n = 71, 30.3%). This was followed by *Escherichia coli* in 33 cases (14.1%) and *Pseudomonas aeruginosa* in 32 cases (13.7%). MDR organisms accounted for 152 infections (65%), presenting a significant treatment challenge. Empiric antibiotic therapy most commonly involved broad-spectrum agents such as imipenem-cilastatin (n= 198, 84.6%) and vancomycin (n= 136, 58.1%). Empiric therapy was deemed appropriate in 111 patients (47.4%), inappropriate in 74 (31.6%), and not assessable due to missing data in 49 (20.9%).

Following culture and susceptibility results, antibiotics in 87 cases (37.2%) remained unchanged. In multivariate analysis, only infection severity, specifically, patients with IWGDF/IDSA Grade 3 or 4 compared to Grade 1 or 2, was significantly associated with prolonged hospitalization (β = -0.161, P = 0.034).”

Missing Data Reporting

Abstract omits the number of patients excluded due to missing data (20.9%). This must be explicitly stated.

Response

Thank you for highlighting this important point. We have revised the Results section of the Abstract to explicitly state the proportion of patients for whom empiric therapy assessment was not possible due to missing data (20.9%). The updated sentence now reads:

"Empiric therapy was deemed appropriate in 111 patients (47.4%), inappropriate in 74 (31.6%), and not assessable due to missing data in 49 (20.9%)."

Lack of Research Gap Justification

The manuscript does not clearly explain the novelty of the study in the Jordanian context or how it adds insight beyond existing literature.

Response

Thank you for your comment. We have revised the Background section to clearly highlight the research gap. Our study is, to our knowledge, the first in Jordan to assess the appropriateness of empirical antibiotic therapy in DFIs and its association with multidrug resistance and length of hospital stay. This adds new insights to the existing local literature.

The added paragraph:

“Although some studies in Jordan have investigated aspects of DFIs, such as pathogen prevalence, resistance patterns, or virulence traits of specific organisms, there remains a lack of comprehensive data evaluating the full microbiological profile, resistance characteristics, and clinical outcomes in a single cohort (10-12). Importantly, no Jordanian studies to date have assessed the appropriateness of empirical antimicrobial therapy in DFI or examined its association with clinical outcomes such as length of hospital stay. Similarly, the impact of multidrug-resistant (MDR) infections on hospitalization duration has not been systematically explored in the local context.
By addressing these gaps, this study aims to generate clinically relevant, locally grounded evidence to inform more effective treatment strategies and guide antimicrobial stewardship efforts in Jordan.”

Empiric Therapy Assessment

It is unclear whether both antimicrobial spectrum coverage and dosing adequacy were systematically evaluated.

Response

Thank you for your comment. We confirm that the assessment of empiric therapy considered both the spectrum of antimicrobial coverage and dosing adequacy. As clarified in the Methods section (Section 2.4 – Study Outcomes), therapy was classified as appropriate only if at least one empiric antibiotic administered within the first 48 hours covered all identified pathogens and was given with the correct dosage, formulation, and route of administration. Cases that did not meet these criteria were considered inappropriate.

Handling of Missing Data

No description is provided for how missing data (20.9% for appropriateness assessment) were addressed statistically.

Response

Thank you for your comment. We acknowledge that 20.9% of cases had missing data regarding the appropriateness of empiric therapy, primarily due to unavailable or incomplete susceptibility results. No statistical imputation or formal handling of missing data was performed in this study. Cases with missing data were excluded from analyses that required this information, such as the assessment of empiric therapy appropriateness, but were included in other analyses when relevant data were available. We have added a statement to the Methods section to clarify this point as separate section 2.6.

### “2.6. Handling of Missing Data: No imputation was performed for missing data. Patients with incomplete or unavailable culture and susceptibility results were excluded from analyses evaluating the appropriateness of empiric antibiotic therapy. These patients were retained in other analyses when relevant data were available.”

Length of Stay (LOS) Definition

It is unclear whether LOS is counted from admission to discharge or only for infection-related days.

Response

Thank you for your observation. We clarify that length of stay (LOS) was defined as the total number of days from hospital admission to discharge, regardless of whether all days were directly related to infection management. This has been added to the Methods section (section 2.4) for clarity as follows:

“The third outcome examined in this study was the duration of hospitalization. Specifically, we assessed how factors such as antimicrobial resistance patterns and the appropriateness of initial antibiotic therapy influenced the length of stay. For this analysis, length of stay was calculated as the total number of days from the patient’s admission to discharge, encompassing the entire hospital stay regardless of whether each day was directly related to infection treatment. In addition to antimicrobial factors, we considered a range of demographic and clinical variables as potential contributors to prolonged hospitalization. These included age, gender, smoking status, type of diabetes, HbA1c levels, department of admission (internal medicine vs. surgical wards), ICU transfer during hospitalization, presence of diabetic foot ulcers, and infection severity based on the IWGDF/IDSA classification.”

No Power/Sample Size Calculation

The study lacks any sample size justification or power analysis.

Response

Thank you for highlighting this point. As this study was retrospective and observational in nature, the sample size was determined by the number of eligible cases available during the study period rather than by a priori power calculation.

This information was added to at the end of the first paragraph of Section 2.1.

Retrospective Design Limitations

Methodological limitations inherent to retrospective design are not discussed. Operational definitions, missing data handling, and rationale for statistical tests must be clarified.

Response

Thank you for your insightful comments. We have addressed each of the points raised as follows:

- **Retrospective design limitations:** We added a detailed discussion in the limitations section acknowledging the retrospective nature of the study and its constraints:
  *“Furthermore, the retrospective design limited the ability to assess the clinical decision-making processes that contributed to empirical therapy choices and subsequent adjustments.”*
- **Operational definitions:** Key terms were explicitly defined in the Methods section:
  *“Therapy was considered appropriate if at least one antibiotic given within the first 48 hours of admission covered all identified pathogens and was administered with the correct dosage, formulation, and route. If any of these factors were lacking, the therapy was deemed inappropriate.”*
  and
  *“Length of stay was calculated as the total number of days from the patient’s admission to discharge, encompassing the entire hospital stay regardless of whether each day was directly related to infection treatment.”*
- **Handling of missing data:** We clearly described our approach to missing data:
  *“No imputation was performed for missing data. Patients with incomplete or unavailable culture and susceptibility results were excluded from analyses evaluating the appropriateness of empiric antibiotic therapy.”*
- **Statistical methods rationale:** The statistical analysis section now explains the tests used and the rationale:

Results Presentation

Results are repetitive, descriptive data in text duplicate tables.

Response

Thank you for your comment. We have addressed the issue of repetition by condensing descriptive text in the results section.

Resistance analysis is superficial and no organism-specific resistance rates are presented. The counts across Abstract, Results, and Tables do not match.

Response

We have carefully reviewed and corrected all counts and percentages across the Abstract, Results, and Tables to ensure internal consistency.

Include a supplementary table with detailed susceptibility profiles per organism and consistent denominators.

Response

Thank you for your comment. We have included a supplementary table providing detailed susceptibility profiles for each organism.

Shapiro–Wilk test is acceptable, but for sample sizes ≥50, tests like Kolmogorov–Smirnov are often preferred. Rationale for choice should be stated. LOS is influenced by severity, comorbidities, ICU stay, etc. Simple two-group comparisons risk bias. Authors should clarify whether these factors were considered. A multivariate linear regression (with log-transformed LOS) or Cox proportional hazards model (for discharge time) would allow adjustment for confounders. I recommend re-running the statistical analysis accordingly.

Response

Thank you for your suggestion. In response, we re-evaluated the distribution of continuous variables using the Kolmogorov–Smirnov test, which is more suitable for larger sample sizes (≥50). The results were consistent with our original assessment using the Shapiro–Wilk test. We have updated the Statistical Analysis section to reflect this change.

We agree that length of stay (LOS) is influenced by multiple clinical factors, and we have addressed this by conducting a multivariate linear regression analysis using log-transformed LOS as the dependent variable to account for skewness.

To adjust for confounding, we included a comprehensive set of clinically relevant variables in the model. These included age, gender, smoking status, type of diabetes, HbA1c levels, department of admission (internal medicine vs surgery department), ICU transfer during hospitalization, presence of diabetic foot ulcers, infection severity based on the IWGDF/IDSA classification, appropriateness of empiric antibiotic therapy, and the presence of MDR infection.

The Results section was updated to include Table 3, and the corresponding text was revised as follows:

“A multivariate linear regression analysis was conducted to identify factors associated with the log-transformed length of hospital stay among patients with DFIs (Table 3). In the univariate analysis, seven predictors had P values less than 0.250, making them eligible for inclusion in the multivariate linear regression model. These included type of diabetes (P = 0.105), department of admission (P = 0.048), ICU transfer (P = 0.011), presence of diabetic foot ulcer (P = 0.051), infection severity based on the IWGDF/IDSA classification (P = 0.015), appropriate empiric therapy (P = 0.234), and presence of MDR infection (P = 0.069).

After adjusting for covariates in the multivariate model, only the severity of infection remained a statistically significant predictor. Patients with less severe infections (IWGDF/IDSA Grader 2) had significantly shorter hospital stays compared to those with more severe infections (Grade 3 or 4; β = -0.161, P = 0.034).”

Discussion Interpretation

Overstates similarity to other studies without considering local microbiology patterns. Does not explore possible causes for high inappropriate empiric therapy rates. LOS is significant for therapy appropriateness but not MDR status and the reasons are not discussed.

Response

Thank you for your valuable feedback. We have carefully addressed the points raised in our revised manuscript as follows:

1. **Local microbiology patterns:**
   We expanded the discussion to emphasize the influence of regional factors on pathogen distribution and resistance patterns:
   *"While Staphylococcus aureus remains a leading pathogen globally, the notable presence of Gram-negative bacteria such as Pseudomonas aeruginosa highlights the importance of considering local microbiological patterns that may differ due to regional factors such as environmental conditions and healthcare practices (26)."*
2. **Causes of high inappropriate empiric therapy rates:**
   We explored potential reasons for the relatively high rate of inappropriate empirical therapy:
   *"Reasons for this may include limited access to timely culture and sensitivity results, lack of updated local antibiograms, and variability in clinical decision-making, especially in the absence of standardized local protocols (33)."*
3. **Length of stay (LOS), therapy appropriateness, and MDR status:**
   We clarified the relationship between LOS and therapy appropriateness, and discussed why MDR status was not significantly associated with prolonged hospitalization:
   *"In this study, neither the appropriateness of empirical antibiotic therapy nor the presence of MDR infections showed a statistically significant association with length of hospital stay. This may be explained by the possibility that early modification of antibiotics after culture results reduced the impact of initial inappropriate therapy, and that hospital stay is more strongly influenced by infection severity and other host factors rather than MDR status alone."*

Integration with Local Context

Discussion should link observed patterns to local clinical practices (e.g., empiric prescribing habits, lab turnaround times).

Response

We have discussed this by stating:

"Reasons for this may include limited access to timely culture and sensitivity results, lack of updated local antibiograms, and variability in clinical decision-making, especially in the absence of standardized local protocols."

This links observed patterns to local clinical practices such as empiric prescribing habits and lab turnaround times.

Reviewer #2: Overall Recommendation: Major Revisions

Overall Impression:

This manuscript presents a valuable retrospective analysis of diabetic foot infections (DFIs) at a tertiary care center in Jordan. The manuscript is generally well-structured. However, major concerns regarding potential selection bias, data analysis, and the interpretation of results must be addressed before the manuscript can be considered for publication.

Detailed Comments:

1. The exclusion of 79.6% of screened cases (911/1145) of screened cases creates significant selection bias, likely over-representing complex infections. Therefore, the pathogen prevalence and MDR rates cannot be considered representative of the center's true DFI population and must be highlighted as a major limitation.

Response

We thank the reviewer for this important observation. We agree that the exclusion of a large proportion of screened cases may have introduced selection bias, potentially affecting the generalizability of our findings, particularly with respect to pathogen prevalence and MDR rates. In response, we have revised the Limitations section to explicitly acknowledge this issue:

We have added this point as follows:

“Additionally, the exclusion of a large proportion of screened cases, primarily those with incomplete microbiological data may have introduced selection bias, potentially overrepresenting more severe or complex infections and affecting the observed pathogen distribution and resistance rates.”

2. The claim that inappropriate empiric therapy independently prolongs hospital stay is not supported by the current analysis. The analysis must account for confounding by infection severity and other clinical factors. A multivariate model is essential to establish this relationship.
Response

Thank you for your valuable suggestion. We agree that length of stay (LOS) is influenced by multiple clinical factors, and we have addressed this by conducting a multivariate linear regression analysis using log-transformed LOS as the dependent variable to account for skewness.

To adjust for confounding, we included a comprehensive set of clinically relevant variables in the model. These included age, gender, smoking status, type of diabetes, HbA1c levels, department of admission (internal medicine vs surgery department), ICU transfer during hospitalization, presence of diabetic foot ulcers, infection severity based on the IWGDF/IDSA classification, appropriateness of empiric antibiotic therapy, and the presence of MDR infection.

The Results section was updated to include Table 3, and the corresponding text was revised as follows:

“A multivariate linear regression analysis was conducted to identify factors associated with the log-transformed length of hospital stay among patients with DFIs (Table 3). In the univariate analysis, seven predictors had P values less than 0.250, making them eligible for inclusion in the multivariate linear regression model. These included type of diabetes (P = 0.105), department of admission (P = 0.048), ICU transfer (P = 0.011), presence of diabetic foot ulcer (P = 0.051), infection severity based on the IWGDF/IDSA classification (P = 0.015), appropriate empiric therapy (P = 0.234), and presence of MDR infection (P = 0.069).

After adjusting for covariates in the multivariate model, only the severity of infection remained a statistically significant predictor. Patients with less severe infections (IWGDF/IDSA Grader 2) had significantly shorter hospital stays compared to those with more severe infections (Grade 3 or 4; β = -0.161, P = 0.034).”

3. There are discrepancies between the Abstract and the main Results section (3.4) that must be reconciled for the manuscript to be accurate and credible.

Response

Thank you for pointing out the discrepancies between the Abstract and the Results section. These inconsistencies were inadvertently introduced. We have carefully reviewed and corrected all relevant data in the result section of the abstract.

a. Abstract: S. aureus = 88 (37.6%); Results: Staphylococcus species = 117 (50%). Please clarify the breakdown between S. aureus (MSSA+MRSA) and other Staphylococci.

Response

Thank you for pointing out the discrepancies between the Abstract and the Results section. These inconsistencies were inadvertently introduced. We have carefully reviewed and corrected all relevant data in the result section of the abstract.

The sentence was revised as follows:

“The most frequently isolated bacterium was Staphylococcus aureus, identified in 117 cases (50.0%), including MRSA (n = 46, 19.6%) and MSSA (n = 71, 30.3%). This was followed by Escherichia coli in 33 cases (14.1%) and Pseudomonas aeruginosa in 32 cases (13.7%).”

b. Abstract: P. aeruginosa = 40 (17.1%); Results: P. aeruginosa = 32 (13.7%).

Response

The sentence was revised as follows:

“The most frequently isolated bacterium was Staphylococcus aureus, identified in 117 cases (50.0%), including MRSA (n = 46, 19.6%) and MSSA (n = 71, 30.3%). This was followed by Escherichia coli in 33 cases (14.1%) and Pseudomonas aeruginosa in 32 cases (13.7%).”

c. Abstract: E. coli = 35 (15.0%); Results: E. coli = 33 (14.1%).

Response

The sentence was revised as follows:

“The most frequently isolated bacterium was Staphylococcus aureus, identified in 117 cases (50.0%), including MRSA (n = 46, 19.6%) and MSSA (n = 71, 30.3%). This was followed by Escherichia coli in 33 cases (14.1%) and Pseudomonas aeruginosa in 32 cases (13.7%).”

Please carefully review and ensure all data points are consistent throughout the manuscript.

Response

Thank you for pointing out the discrepancies between the Abstract and the Results section. These inconsistencies were inadvertently introduced. We have carefully reviewed and corrected all relevant data in the result section of the abstract.

4. The flowchart (Figure 2) is currently confusing and difficult to follow. The numbers in the boxes (e.g., "29 received appropriate...") do not match the totals in the text. This figure should be redesigned for clarity to accurately visually represent the patient flow from screening through the assessment of empiric and definitive therapy.

Response

Thank you for your observation. Figure 2 has been revised for clarity and now accurately reflects the patient flow and the numbers reported in the text. Specifically, the total number of patients who received appropriate empiric therapy is correctly shown as 111 (29 discharged before culture results + 82 remained hospitalized), consistent with the main text. We appreciate your feedback in helping improve the figure’s clarity.

5. Please define "concurrent infections" in the exclusion criteria. What types of infections were excluded (e.g., pneumonia, UTI)?

Response

Thank you for your comment. We have clarified the exclusion criteria by defining "concurrent infections" as infections other than diabetic foot infections that were present during the same hospitalization and could confound the analysis. These included infections such as pneumonia, urinary tract infections, bloodstream infections, and other systemic or localized infections. This clarification has been added to the Methods section under exclusion criteria as follows:

“Patients were excluded if they lacked susceptibility testing results, had no available or negative culture results, were managed as outpatients, or had concurrent infections (any other active infections during hospitalization, such as pneumonia, urinary tract infections, bloodstream infections, or other systemic or localized infections that could affect clinical outcomes).
