# Supplementary material for: Multidrug resistance and inappropriate empiric therapy as predictors of hospital stay in diabetic foot infections
Source: PLoS One. 2026 Jun 8;21(6):e0350837. doi: 10.1371/journal.pone.0350837 (PMC13245766; doi:10.1371/journal.pone.0350837)
Supplement: Attachment — Submitted filename: PLOS one my reviewer comments.docx [file pone.0350837.s005.docx]

Dear editor,

This manuscript addresses an important and clinically relevant topic: the microbiology, antimicrobial resistance patterns, appropriateness of empiric therapy, and length of hospital stay among patients with diabetic foot infections (DFIs). The study uses a relatively large sample, covers nearly a decade of data, and provides valuable insight into the empirical treatment practices in a tertiary center in Jordan. Overall, the topic is suitable for PLOS ONE, and the manuscript has publication potential. However, several methodological, analytical, and presentation-related issues require substantial revision before the paper can be considered for acceptance.

The main concern relates to the clarity and consistency of the definitions used in the study—especially the criteria for "appropriate" empiric therapy. The manuscript states that therapy was considered appropriate if at least one antibiotic administered in the first 48 hours covered all isolated pathogens with correct dose, route, and formulation. However, this definition lacks operational detail. It remains unclear how treatment adequacy was judged in polymicrobial infections, how dosing appropriateness was evaluated, and what specific criteria led to classifying 49 cases as “insufficient data.” A more explicit, standardized definition—ideally presented in a table—would greatly enhance reproducibility and transparency.

There is also an issue with the MDR definition. Although the manuscript uses a definition based on resistance to at least one agent in three or more antimicrobial classes, the cited reference does not correspond to the widely accepted Magiorakos et al. (2012, Clinical Microbiology and Infection) framework. This internationally recognized definition should be used and appropriately referenced.

The statistical analysis is descriptive and univariate, relying primarily on Mann–Whitney U tests. Length of stay is influenced by multiple potential confounders, including age, infection severity, comorbidities, presence of osteomyelitis, ICU admission, and number of pathogens isolated. Without a multivariable regression model, the conclusion that inappropriate empiric therapy prolongs hospitalization remains incomplete. A regression approach—linear, Poisson, or negative binomial, depending on LOS distribution—would substantially strengthen the findings.

The flow diagram presented in the manuscript is somewhat confusing. Excluded cases are described narratively but are not shown visually. The diagram would benefit from a clearer PRISMA-style structure detailing screened cases, exclusions with reasons, and final analytic cohorts.

The discussion section provides an overview of relevant literature but would benefit from deeper engagement with the study’s own findings. In particular, the lack of a statistically significant association between MDR infections and length of stay warrants a more nuanced explanation. The sustained use of broad-spectrum therapy despite availability of culture results is important and could be explored more thoroughly in terms of stewardship implications, local prescribing culture, and institutional factors.

Several tables and figures require clearer formatting, more descriptive legends, and more precise labeling. Some variables in Tables 1 and 2 are presented in ways that may confuse readers, and percentages sometimes do not sum intuitively. Presenting the transitions between empiric and definitive therapy in a table (rather than solely in the text) would also improve clarity.

Minor issues include occasional repetition, slight inconsistencies in terminology, and referencing concerns. The manuscript is generally well written, but some sections—especially the introduction and discussion—could be more concise. The abstract could be shortened for clarity. Microorganism names should be written in italic. It should be corrected in all sections.

Ethical considerations are adequately addressed, although the data availability statement may require refinement to fully comply with PLOS ONE expectations. It may help to clarify why anonymization alone is insufficient to meet public data-sharing requirements.

In its current form, the manuscript contributes valuable descriptive epidemiology but requires substantial methodological refinement and clarification. With careful revision, including clearer definitions, improved statistical modeling, more structured data presentation, and deeper interpretation, the study would be suitable for reconsideration.
