# Supplementary material for: Multidrug resistance and inappropriate empiric therapy as predictors of hospital stay in diabetic foot infections
Source: PLoS One. 2026 Jun 8;21(6):e0350837. doi: 10.1371/journal.pone.0350837 (PMC13245766; doi:10.1371/journal.pone.0350837)
Supplement: Attachment — Submitted filename: Reply_to_Reviewers_auresp_2.docx [file pone.0350837.s007.docx]

Review Comments to the Author

Reviewer #3: Dear author (s)
I have some revision suggestions below for your manuscript.
The main concern relates to the clarity and consistency of the definitions used in the study—especially the criteria for "appropriate" empiric therapy. The manuscript states that therapy was considered appropriate if at least one antibiotic administered in the first 48 hours covered all isolated pathogens with correct dose, route, and formulation. However, this definition lacks operational detail. It remains unclear how treatment adequacy was judged in polymicrobial infections, how dosing appropriateness was evaluated, and what specific criteria led to classifying 49 cases as “insufficient data.” A more explicit, standardized definition—ideally presented in a table—would greatly enhance reproducibility and transparency.

Response

We agree with the reviewer’s assessment and have revised Section 2.4 to provide a standardized operational definition based on contemporary literature (Van Heuverswyn et al., 2023). Specifically, we adopted a 'strict-concordance' model where appropriateness required 100% coverage of all isolates in polymicrobial infections, correct renal dosing, and administration within 48 hours. As suggested, we have added **Table 1** to clearly outline the decision matrix used for these classifications and to detail the exclusion criteria for the 49 'insufficient data' cases."

**Table 1.** Operational Definitions for Empirical Antibiotic Appropriateness

| Category | Criteria for "Appropriate" Classification |
| --- | --- |
| Microbiological Concordance | All isolated pathogens must be susceptible *in vitro* to at least one empirical agent. In polymicrobial infections, 100% of isolates must be covered (15). |
| Dosing & Route | Dose, formulation, and route must align with IWGDF/IDSA 2024 standards, adjusted for admission renal function (12). |
| Timing | First dose must be administered within 48 hours of hospital admission. |
| Insufficient Data | Assigned when susceptibility results were incomplete for any isolate or when medication administration timestamps were missing from the electronic record. |

There is also an issue with the MDR definition. Although the manuscript uses a definition based on resistance to at least one agent in three or more antimicrobial classes, the cited reference does not correspond to the widely accepted Magiorakos et al. (2012, Clinical Microbiology and Infection) framework. This internationally recognized definition should be used and appropriately referenced.

Response

We thank the reviewer for this important comment. We have revised the manuscript to adopt the internationally accepted definition of multidrug resistance as proposed by Magiorakos et al. (2012, Clinical Microbiology and Infection), and the previous reference has been replaced accordingly.

The statistical analysis is descriptive and univariate, relying primarily on Mann–Whitney U tests. Length of stay is influenced by multiple potential confounders, including age, infection severity, comorbidities, presence of osteomyelitis, ICU admission, and number of pathogens isolated. Without a multivariable regression model, the conclusion that inappropriate empiric therapy prolongs hospitalization remains incomplete. A regression approach—linear, Poisson, or negative binomial, depending on LOS distribution—would substantially strengthen the findings.

Response

We agree that length of stay (LOS) is influenced by multiple clinical factors, and we have addressed this by conducting a multivariate linear regression analysis using log-transformed LOS as the dependent variable to account for skewness.

To adjust for confounding, we included a comprehensive set of clinically relevant variables in the model. These included age, gender, smoking status, type of diabetes, HbA1c levels, department of admission (internal medicine vs surgery department), ICU transfer during hospitalization, presence of diabetic foot ulcers, infection severity based on the IWGDF/IDSA classification, appropriateness of empiric antibiotic therapy, and the presence of MDR infection.

The Results section was updated to include Table 3, and the corresponding text was revised as follows:

“A multivariate linear regression analysis was conducted to identify factors associated with the log-transformed length of hospital stay among patients with DFIs (Table 3). In the univariate analysis, seven predictors had P values less than 0.250, making them eligible for inclusion in the multivariate linear regression model. These included type of diabetes (P = 0.105), department of admission (P = 0.048), ICU transfer (P = 0.011), presence of diabetic foot ulcer (P = 0.051), infection severity based on the IWGDF/IDSA classification (P = 0.015), appropriate empiric therapy (P = 0.234), and presence of MDR infection (P = 0.069).

After adjusting for covariates in the multivariate model, only the severity of infection remained a statistically significant predictor. Patients with less severe infections (IWGDF/IDSA Grader 2) had significantly shorter hospital stays compared to those with more severe infections (Grade 3 or 4; β = -0.161, P = 0.034).”

The flow diagram presented in the manuscript is somewhat confusing. Excluded cases are described narratively but are not shown visually. The diagram would benefit from a clearer PRISMA-style structure detailing screened cases, exclusions with reasons, and final analytic cohorts.

Response

We thank the reviewer for this constructive suggestion. We agree that a visual representation of the screening and exclusion process enhances the clarity of the study’s methodology.

In response, we have revised Figure 1 to follow a PRISMA-style format. The updated figure now explicitly illustrates the flow from the initial identification of cases (n=1,145) to the final analytic cohort (n=234). Specifically, we have added an exclusion box that details the 911 cases excluded, categorized by reason (lack of adequate data, negative culture results, outpatient management, and concurrent infections). We have also streamlined the visual elements and added phase labels (Identification, Screening and Exclusions, and Analysis) to meet standard journal reporting requirements. We believe this revision provides a more transparent and comprehensive overview of our study population.

The discussion section provides an overview of relevant literature but would benefit from deeper engagement with the study’s own findings. In particular, the lack of a statistically significant association between MDR infections and length of stay warrants a more nuanced explanation. The sustained use of broad-spectrum therapy despite availability of culture results is important and could be explored more thoroughly in terms of stewardship implications, local prescribing culture, and institutional factors.

Response

We thank the reviewer for this constructive critique. The Discussion section has been extensively revised to provide a deeper, more nuanced engagement with our primary findings. Specifically, we have added an analysis of the 'neutralization effect,' where aggressive early empirical therapy may have mitigated the expected impact of MDR pathogens on hospitalization duration. Furthermore, we have introduced a critical exploration of 'clinical inertia' and institutional stewardship barriers—such as the lack of formalized multidisciplinary 'antibiotic timeouts'—to explain the sustained use of broad-spectrum therapy despite culture availability

Several tables and figures require clearer formatting, more descriptive legends, and more precise labeling. Some variables in Tables 1 and 2 are presented in ways that may confuse readers, and percentages sometimes do not sum intuitively. Presenting the transitions between empiric and definitive therapy in a table (rather than solely in the text) would also improve clarity.

Response

All tables and figures have been revised for improved clarity and precision. Variable labeling and legends were updated to ensure technical accuracy, and all percentages were audited and corrected to ensure intuitive summation. As suggested, a new table (Table 4) has been added to clearly present the transitions from empirical to definitive therapy

Minor issues include occasional repetition, slight inconsistencies in terminology, and referencing concerns. The manuscript is generally well written, but some sections—especially the introduction and discussion—could be more concise.

Response

We thank the reviewer for this comment. The manuscript has been carefully revised to reduce repetition, ensure consistency in terminology, and address referencing concerns. The Introduction and Discussion sections have also been refined for improved clarity and conciseness.

The abstract could be shortened for clarity.

Response

The abstract has been shortened for improved clarity and focus.

Microorganism names should be written in italic. It should be corrected in all sections.

Response

All microorganism names have been revised and formatted in italics throughout the manuscript.

Ethical considerations are adequately addressed, although the data availability statement may require refinement to fully comply with PLOS ONE expectations.

Response

We thank the reviewer for this comment. The Data Availability Statement has been revised to fully comply with PLOS ONE requirements by clearly specifying the restrictions on data sharing and the process for accessing the data through the Institutional Review Board of Jordan University Hospital.

It may help to clarify why anonymization alone is insufficient to meet public data-sharing requirements.

Response

We thank the reviewer for this valuable suggestion. We have clarified that anonymization alone is insufficient in this study due to the inclusion of detailed clinical and hospitalization data, which may carry a risk of indirect patient re-identification. Therefore, access to the dataset is restricted and subject to Institutional Review Board approval to ensure compliance with ethical and confidentiality standards.

In its current form, the manuscript contributes valuable descriptive epidemiology but requires substantial methodological refinement and clarification. With careful revision, including clearer definitions, improved statistical modeling, more structured data presentation, and deeper interpretation, the study would be suitable for reconsideration.

Response

We thank the reviewer for identifying areas for improvement. We have revised the manuscript to ensure high methodological rigor. Specifically, we:

- Operationalized Definitions: Adopted a strict 'all-or-nothing' concordance model for antibiotic appropriateness (Van Heuverswyn et al., 2023) and added Table 1 for transparency.
- Strengthened Statistics: Implemented log-transformation for Length of Stay (LOS) in our regression models to correct for data skewness.
- Clarified Exclusions: Defined specific criteria for the 49 'insufficient data' cases to prevent selection bias.
- Enhanced Structure: Reorganized data presentation to ensure all clinical and microbiological outcomes are logically linked to our study objectives.

Reviewer #4: The author(s) have adequately addressed the comments raised in the previous review. However, the following needs to be addressed as well:

RESULTS
Table 1:

Comment:
•Ensure open and closed brackets around the percentages under the Frequency column

Response

All percentages under the Frequency column have been checked, and the use of open and closed brackets has been corrected throughout the manuscript.

•Correct the proportions/percentages of the following categories:
Most common illnesses – Correct the proportion for Chronic kidney failure – it is not 26%.

Response

The proportion for chronic kidney failure under the “Most common illnesses” category has been reviewed and corrected to 22.2% in the revised manuscript.

•Was the patient transferred to ICU – Correct the proportion of ‘No’ – it is not 84.3%

Response

The proportion of patients who were not transferred to the ICU has been reviewed and corrected to 84.6% in the revised manuscript.

3.4 Isolated Bacteria.
Correct the percentage of Enteroccocus spp - 51(51.8%) to the nearest decimal point.

Response

We thank the reviewer for this comment. The percentage of Enterococcus spp. has been reviewed and corrected to 21.8% in the revised manuscript.

3.5 Empirical and Definitive Antimicrobial Therapy
Correct the percentage of ‘Most antibiotics were administered intravenously (n-179; 76.5%) to the nearest decimal point.

Response

The percentage for “Most antibiotics were administered intravenously” has been reviewed and confirmed to be correct at 76.5% in the revised manuscript.

Line 265: You state ‘Following culture and susceptibility results, antibiotics were modified in several ways’. Comment: This text implies that antibiotics were modified, which is not the case. I suppose what was modified was the antibiotic therapy.
Response

We thank the reviewer for this comment. The text has been revised to clarify that “antibiotic therapy,” rather than “antibiotics,” was modified following culture and susceptibility results.

Fig 2 – The flow of data is confusing after the 3rd level category. The implication is that the numbers in the box below a category are a subset of that category, which is not the case.
Response

We appreciate the reviewer’s feedback regarding the visual clarity of the data flow. We have redesigned Figure 2 to follow a standard PRISMA-style branching logic. The vertical 'stacking' has been replaced with horizontal branching to explicitly show that categories (Appropriate, Inappropriate, and Inadequate data) are mutually exclusive subsets of the same parent cohort.

Line 300: You state ‘#using simple linear regression’. For clarity, I suggest you state ‘Using univariable linear regression’ to mean you regressed each variable separately.
Response

We thank the reviewer for this comment. The text has been revised to “using univariate linear regression” to clarify that each variable was analyzed separately.

Line 305: You state ‘This study explored the epidemiology, causative pathogens, resistance patterns, and treatment approaches for DFI at a tertiary care center in Jordan.’
Comment: Epidemiology is a quite broad concept and appears here in the discussion without any reference to it in the title or aim of your study.
Response

We thank the reviewer for this comment. We have revised the sentence to avoid the use of the term “epidemiology,” as it was not explicitly defined as an aim of the study. The text has been modified accordingly to better align with the study objectives as follows:

“This study explored the causative pathogens, antimicrobial resistance patterns, and treatment approaches for DFIs at a tertiary care center in Jordan.”

Line 306-308: You state ‘A total of 234 patients participated, with 307 males representing 75.6% (n=177) and females 24.4% (n=57), emphasizing sex differences in DFI incidence and management (20)’. Comment: A difference should be supported with a p-value showing significance.
Response

We thank the reviewer for this comment. We have revised the sentence by removing the interpretation of sex differences, as no statistical comparison was performed to assess significance between males and females in the study.

Line 366: You state ‘The sample size limited statistical power for subgroup analyses, especially for MDR infections. Comment: It would be desirable to show the post-hoc power of this study based on the sample size used. This comment was also raised previously.

Response
We thank the reviewer for this important comment. Based on the final multivariate regression model (R² = 0.071, f² = 0.076), a post-hoc power analysis indicated that the study achieved an overall statistical power of approximately 0.88 at an alpha level of 0.05. We acknowledge that, despite adequate overall power, subgroup analyses—particularly for MDR infections—may still be underpowered due to limited sample size.

The limitation was revised as follows:

“A post hoc analysis shows the overall regression model has limited explanatory power (R2 = 0.071) but still had acceptable overall statistical power. However, subgroup analyses are still probably underpowered. Consequently, caution must be taken when interpreting these results”

Line 378: Spelling of Klebsiella pneumoniae should be corrected

Response

The spelling of *Klebsiella pneumoniae* has been corrected in the revised manuscript.
