# Supplementary material for: Multidrug resistance and inappropriate empiric therapy as predictors of hospital stay in diabetic foot infections
Source: PLoS One. 2026 Jun 8;21(6):e0350837. doi: 10.1371/journal.pone.0350837 (PMC13245766; doi:10.1371/journal.pone.0350837)
Supplement: Peer review history

# Peer review: Decision Letter 0

**DOI:** 10.1371/journal.pone.0350837.r001

**Authors:** Muhammad Atif

1 Sep 2025

PONE-D-25-38915

Multidrug Resistance and Inappropriate Empiric Therapy as Predictors of Hospital Stay in Diabetic Foot Infections

PLOS ONE

Dear Dr. Itani,

Thank you for submitting your manuscript to PLOS ONE. After careful consideration, we feel that it has merit but does not fully meet PLOS ONE’s publication criteria as it currently stands. Therefore, we invite you to submit a revised version of the manuscript that addresses the points raised during the review process.

Please submit your revised manuscript by Oct 16 2025 11:59PM. If you will need more time than this to complete your revisions, please reply to this message or contact the journal office at plosone@plos.org. When you're ready to submit your revision, log on to https://www.editorialmanager.com/pone/ and select the 'Submissions Needing Revision' folder to locate your manuscript file.

Please include the following items when submitting your revised manuscript:

- A rebuttal letter that responds to each point raised by the academic editor and reviewer(s). You should upload this letter as a separate file labeled 'Response to Reviewers'.
- A marked-up copy of your manuscript that highlights changes made to the original version. You should upload this as a separate file labeled 'Revised Manuscript with Track Changes'.
- An unmarked version of your revised paper without tracked changes. You should upload this as a separate file labeled 'Manuscript'.

If you would like to make changes to your financial disclosure, please include your updated statement in your cover letter. Guidelines for resubmitting your figure files are available below the reviewer comments at the end of this letter.

If applicable, we recommend that you deposit your laboratory protocols in protocols.io to enhance the reproducibility of your results. Protocols.io assigns your protocol its own identifier (DOI) so that it can be cited independently in the future. For instructions see: https://journals.plos.org/plosone/s/submission-guidelines#loc-laboratory-protocols. Additionally, PLOS ONE offers an option for publishing peer-reviewed Lab Protocol articles, which describe protocols hosted on protocols.io. Read more information on sharing protocols at https://plos.org/protocols?utm_medium=editorial-email&utm_source=authorletters&utm_campaign=protocols.

We look forward to receiving your revised manuscript.

Kind regards,

Muhammad Atif

Academic Editor

PLOS ONE

Journal Requirements:

When submitting your revision, we need you to address these additional requirements.

1. Please ensure that your manuscript meets PLOS ONE's style requirements, including those for file naming. The PLOS ONE style templates can be found at

https://journals.plos.org/plosone/s/file?id=wjVg/PLOSOne_formatting_sample_main_body.pdf and

https://journals.plos.org/plosone/s/file?id=ba62/PLOSOne_formatting_sample_title_authors_affiliations.pdf

If the reviewer comments include a recommendation to cite specific previously published works, please review and evaluate these publications to determine whether they are relevant and should be cited. There is no requirement to cite these works unless the editor has indicated otherwise.

[Note: HTML markup is below. Please do not edit.]

Reviewers' comments:

Reviewer's Responses to Questions

**Comments to the Author**

1. Is the manuscript technically sound, and do the data support the conclusions?

The manuscript must describe a technically sound piece of scientific research with data that supports the conclusions. Experiments must have been conducted rigorously, with appropriate controls, replication, and sample sizes. The conclusions must be drawn appropriately based on the data presented.

Reviewer #1: No

Reviewer #2: Yes

**********

2. Has the statistical analysis been performed appropriately and rigorously?

Reviewer #1: No

Reviewer #2: Yes

**********

3. Have the authors made all data underlying the findings in their manuscript fully available?

The PLOS Data policy requires authors to make all data underlying the findings described in their manuscript fully available without restriction, with rare exception (please refer to the Data Availability Statement in the manuscript PDF file). The data should be provided as part of the manuscript or its supporting information, or deposited to a public repository. For example, in addition to summary statistics, the data points behind means, medians and variance measures should be available. If there are restrictions on publicly sharing data—e.g. participant privacy or use of data from a third party—those must be specified.

Reviewer #1: Yes

Reviewer #2: Yes

**********

4. Is the manuscript presented in an intelligible fashion and written in standard English?

PLOS ONE does not copyedit accepted manuscripts, so the language in submitted articles must be clear, correct, and unambiguous. Any typographical or grammatical errors should be corrected at revision, so please note any specific errors here.

Reviewer #1: No

Reviewer #2: Yes

**********

5. Review Comments to the Author

Please use the space provided to explain your answers to the questions above. You may also include additional comments for the author, including concerns about dual publication, research ethics, or publication ethics. (Please upload your review as an attachment if it exceeds 20,000 characters)

Reviewer #1: The manuscript addresses an important and clinically relevant question. However, it has substantial methodological and reporting deficiencies that compromise data integrity, clarity, and policy compliance. Key problems include inconsistencies in reported results, unclear definitions, insufficient statistical rigor, and inadequate discussion of limitations.

I recommend major revision (bordering on rejection unless the authors can perform substantial re-analysis and fully reconcile inconsistencies). Without these changes, the study’s validity and interpretability remain doubtful.

Reviewer #2: Overall Recommendation: Major Revisions

Overall Impression:

This manuscript presents a valuable retrospective analysis of diabetic foot infections (DFIs) at a tertiary care center in Jordan. The manuscript is generally well-structured. However, major concerns regarding potential selection bias, data analysis, and the interpretation of results must be addressed before the manuscript can be considered for publication.

Detailed Comments:

1. The exclusion of 79.6% of screened cases (911/1145) of screened cases creates significant selection bias, likely over-representing complex infections. Therefore, the pathogen prevalence and MDR rates cannot be considered representative of the center's true DFI population and must be highlighted as a major limitation.

2. The claim that inappropriate empiric therapy independently prolongs hospital stay is not supported by the current analysis. The analysis must account for confounding by infection severity and other clinical factors. A multivariate model is essential to establish this relationship.

3. There are discrepancies between the Abstract and the main Results section (3.4) that must be reconciled for the manuscript to be accurate and credible.

a. Abstract: S. aureus = 88 (37.6%); Results: Staphylococcus species = 117 (50%). Please clarify the breakdown between S. aureus (MSSA+MRSA) and other Staphylococci.

b. Abstract: P. aeruginosa = 40 (17.1%); Results: P. aeruginosa = 32 (13.7%).

c. Abstract: E. coli = 35 (15.0%); Results: E. coli = 33 (14.1%).

Please carefully review and ensure all data points are consistent throughout the

4. The flowchart (Figure 2) is currently confusing and difficult to follow. The numbers in the boxes (e.g., "29 received appropriate...") do not match the totals in the text. This figure should be redesigned for clarity to accurately visually represent the patient flow from screening through the assessment of empiric and definitive therapy.

5. Please define "concurrent infections" in the exclusion criteria. What types of infections were excluded (e.g., pneumonia, UTI)?

**********

6. PLOS authors have the option to publish the peer review history of their article (what does this mean?). If published, this will include your full peer review and any attached files.

If you choose “no”, your identity will remain anonymous but your review may still be made public.

**Do you want your identity to be public for this peer review?** For information about this choice, including consent withdrawal, please see our Privacy Policy.

Reviewer #1: **Yes:** Fahad Saleem

Reviewer #2: No

**********

[NOTE: If reviewer comments were submitted as an attachment file, they will be attached to this email and accessible via the submission site. Please log into your account, locate the manuscript record, and check for the action link "View Attachments". If this link does not appear, there are no attachment files.]

While revising your submission, please upload your figure files to the Preflight Analysis and Conversion Engine (PACE) digital diagnostic tool, https://pacev2.apexcovantage.com/. PACE helps ensure that figures meet PLOS requirements. To use PACE, you must first register as a user. Registration is free. Then, login and navigate to the UPLOAD tab, where you will find detailed instructions on how to use the tool. If you encounter any issues or have any questions when using PACE, please email PLOS at figures@plos.org. Please note that Supporting Information files do not need this step.

Attachment Submitted filename: Comments PloS One.docx

Attachment Submitted filename: Recommendation_AY.docx

# Author comment: Author response to Decision Letter 1

**DOI:** 10.1371/journal.pone.0350837.r002

19 Sep 2025

Reviewer #1:

General Comments

The manuscript addresses an important and clinically relevant question. However, it has substantial methodological and reporting deficiencies that compromise data integrity, clarity, and policy compliance. Key problems include inconsistencies in reported results, unclear definitions, insufficient statistical rigor, and inadequate discussion of limitations.

I recommend major revision (bordering on rejection unless the authors can perform substantial re-analysis and fully reconcile inconsistencies). Without these changes, the study’s validity and interpretability remain doubtful.

Major Issues

Core Data Inconsistency

Multiple discrepancies between Abstract and Results (e.g., S. aureus in Abstract: 88 cases [37.6%] vs. Staphylococcus spp. in Results: 117 [50%]). LOS reported as mean in Abstract vs. median in Results. These inconsistencies occur throughout the paper. All counts, percentages, and summary measures must be reconciled and harmonized.

Response

Thank you for pointing out the discrepancies between the Abstract and the Results section. These inconsistencies were inadvertently introduced. We have carefully reviewed and corrected all relevant data in the result section of the abstract as follows:

“Results: The most frequently isolated bacterium was Staphylococcus aureus, identified in 117 cases (50.0%), including MRSA (n = 46, 19.6%) and MSSA (n = 71, 30.3%). This was followed by Escherichia coli in 33 cases (14.1%) and Pseudomonas aeruginosa in 32 cases (13.7%). MDR organisms accounted for 152 infections (65%), presenting a significant treatment challenge. Empiric antibiotic therapy most commonly involved broad-spectrum agents such as imipenem-cilastatin (n= 198, 84.6%) and vancomycin (n= 136, 58.1%). Empiric therapy was deemed appropriate in 111 patients (47.4%), inappropriate in 74 (31.6%), and not assessable due to missing data in 49 (20.9%).

Following culture and susceptibility results, antibiotics in 87 cases (37.2%) remained unchanged. In multivariate analysis, only infection severity, specifically, patients with IWGDF/IDSA Grade 3 or 4 compared to Grade 1 or 2, was significantly associated with prolonged hospitalization (β = -0.161, P = 0.034).”

Missing Data Reporting

Abstract omits the number of patients excluded due to missing data (20.9%). This must be explicitly stated.

Response

Thank you for highlighting this important point. We have revised the Results section of the Abstract to explicitly state the proportion of patients for whom empiric therapy assessment was not possible due to missing data (20.9%). The updated sentence now reads:

"Empiric therapy was deemed appropriate in 111 patients (47.4%), inappropriate in 74 (31.6%), and not assessable due to missing data in 49 (20.9%)."

Lack of Research Gap Justification

The manuscript does not clearly explain the novelty of the study in the Jordanian context or how it adds insight beyond existing literature.

Response

Thank you for your comment. We have revised the Background section to clearly highlight the research gap. Our study is, to our knowledge, the first in Jordan to assess the appropriateness of empirical antibiotic therapy in DFIs and its association with multidrug resistance and length of hospital stay. This adds new insights to the existing local literature.

The added paragraph:

“Although some studies in Jordan have investigated aspects of DFIs, such as pathogen prevalence, resistance patterns, or virulence traits of specific organisms, there remains a lack of comprehensive data evaluating the full microbiological profile, resistance characteristics, and clinical outcomes in a single cohort (10-12). Importantly, no Jordanian studies to date have assessed the appropriateness of empirical antimicrobial therapy in DFI or examined its association with clinical outcomes such as length of hospital stay. Similarly, the impact of multidrug-resistant (MDR) infections on hospitalization duration has not been systematically explored in the local context.

By addressing these gaps, this study aims to generate clinically relevant, locally grounded evidence to inform more effective treatment strategies and guide antimicrobial stewardship efforts in Jordan.”

Empiric Therapy Assessment

It is unclear whether both antimicrobial spectrum coverage and dosing adequacy were systematically evaluated.

Response

Thank you for your comment. We confirm that the assessment of empiric therapy considered both the spectrum of antimicrobial coverage and dosing adequacy. As clarified in the Methods section (Section 2.4 – Study Outcomes), therapy was classified as appropriate only if at least one empiric antibiotic administered within the first 48 hours covered all identified pathogens and was given with the correct dosage, formulation, and route of administration. Cases that did not meet these criteria were considered inappropriate.

Handling of Missing Data

No description is provided for how missing data (20.9% for appropriateness assessment) were addressed statistically.

Response

Thank you for your comment. We acknowledge that 20.9% of cases had missing data regarding the appropriateness of empiric therapy, primarily due to unavailable or incomplete susceptibility results. No statistical imputation or formal handling of missing data was performed in this study. Cases with missing data were excluded from analyses that required this information, such as the assessment of empiric therapy appropriateness, but were included in other analyses when relevant data were available. We have added a statement to the Methods section to clarify this point as separate section 2.6.

“2.6. Handling of Missing Data: No imputation was performed for missing data. Patients with incomplete or unavailable culture and susceptibility results were excluded from analyses evaluating the appropriateness of empiric antibiotic therapy. These patients were retained in other analyses when relevant data were available.”

Length of Stay (LOS) Definition

It is unclear whether LOS is counted from admission to discharge or only for infection-related days.

Response

Thank you for your observation. We clarify that length of stay (LOS) was defined as the total number of days from hospital admission to discharge, regardless of whether all days were directly related to infection management. This has been added to the Methods section (section 2.4) for clarity as follows:

“The third outcome examined in this study was the duration of hospitalization. Specifically, we assessed how factors such as antimicrobial resistance patterns and the appropriateness of initial antibiotic therapy influenced the length of stay. For this analysis, length of stay was calculated as the total number of days from the patient’s admission to discharge, encompassing the entire hospital stay regardless of whether each day was directly related to infection treatment. In addition to antimicrobial factors, we considered a range of demographic and clinical variables as potential contributors to prolonged hospitalization. These included age, gender, smoking status, type of diabetes, HbA1c levels, department of admission (internal medicine vs. surgical wards), ICU transfer during hospitalization, presence of diabetic foot ulcers, and infection severity based on the IWGDF/IDSA classification.”

No Power/Sample Size Calculation

The study lacks any sample size justification or power analysis.

Response

Thank you for highlighting this point. As this study was retrospective and observational in nature, the sample size was determined by the number of eligible cases available during the study period rather than by a priori power calculation.

This information was added to at the end of the first paragraph of Section 2.1.

Retrospective Design Limitations

Methodological limitations inherent to retrospective design are not discussed. Operational definitions, missing data handling, and rationale for statistical tests must be clarified.

Response

Thank you for your insightful comments. We have addressed each of the points raised as follows:

• Retrospective design limitations: We added a detailed discussion in the limitations section acknowledging the retrospective nature of the study and its constraints:

“Furthermore, the retrospective design limited the ability to assess the clinical decision-making processes that contributed to empirical therapy choices and subsequent adjustments.”

• Operational definitions: Key terms were explicitly defined in the Methods section:

“Therapy was considered appropriate if at least one antibiotic given within the first 48 hours of admission covered all identified pathogens and was administered with the correct dosage, formulation, and route. If any of these factors were lacking, the therapy was deemed inappropriate.”

and

“Length of stay was calculated as the total number of days from the patient’s admission to discharge, encompassing the entire hospital stay regardless of whether each day was directly related to infection treatment.”

• Handling of missing data: We clearly described our approach to missing data:

“No imputation was performed for missing data. Patients with incomplete or unavailable culture and susceptibility results were excluded from analyses evaluating the appropriateness of empiric antibiotic therapy.”

• Statistical methods rationale: The statistical analysis section now explains the tests used and the rationale:

Results Presentation

Results are repetitive, descriptive data in text duplicate tables.

Response

Thank you for your comment. We have addressed the issue of repetition by condensing descriptive text in the results section.

Resistance analysis is superficial and no organism-specific resistance rates are presented. The counts across Abstract, Results, and Tables do not match.

Response

We have carefully reviewed and corrected all counts and percentages across the Abstract, Results, and Tables to ensure internal consistency.

Include a supplementary table with detailed susceptibility profiles per organism and consistent denominators.

Response

Thank you for your comment. We have included a supplementary table providing detailed susceptibility profiles for each organism.

Shapiro–Wilk test is acceptable, but for sample sizes ≥50, tests like Kolmogorov–Smirnov are often preferred. Rationale for choice should be stated. LOS is influenced by severity, comorbidities, ICU stay, etc. Simple two-group comparisons risk bias. Authors should clarify whether these factors were considered. A multivariate linear regression (with log-transformed LOS) or Cox proportional hazards model (for discharge time) would allow adjustment for confounders. I recommend re-running the statistical analysis accordingly.

Response

Thank you for your suggestion. In response, we re-evaluated the distribution of continuous variables using the Kolmogorov–Smirnov test, which is more suitable for larger sample sizes (≥50). The results were consistent with our original assessment using the Shapiro–Wilk test. We have updated the Statistical Analysis section to reflect this change.

We agree that length of stay (LOS) is influenced by multiple clinical factors, and we have addressed this by conducting a multivariate linear regression analysis using log-transformed LOS as the dependent variable to account for skewness.

To adjust for confounding, we included a comprehensive set of clinically relevant variables in the model. These included age, gender, smoking status, type of diabetes, HbA1c levels, department of admission (internal medicine vs surgery department), ICU transfer during hospitalization, presence of diabetic foot ulcers, infection severity based on the IWGDF/IDSA classification, appropriateness of empiric antibiotic therapy, and the presence of MDR infection.

The Results section was updated to include Table 3, and the corresponding text was revised as follows:

“A multivariate linear regression analysis was conducted to identify factors associated with the log-transformed length of hospital stay among patients with DFIs (Table 3). In the univariate analysis, seven predictors had P values less than 0.250, making them eligible for inclusion in the multivariate linear regression model. These included type of diabetes (P = 0.105), department of admission (P = 0.048), ICU transfer (P = 0.011), presence of diabetic foot ulcer (P = 0.051), infection severity based on the IWGDF/IDSA classification (P = 0.015), appropriate empiric therapy (P = 0.234), and presence of MDR infection (P = 0.069).

After adjusting for covariates in the multivariate model, only the severity of infection remained a statistically significant predictor. Patients with less severe infections (IWGDF/IDSA Grader 2) had significantly shorter hospital stays compared to those with more severe infections (Grade 3 or 4; β = -0.161, P = 0.034).”

Discussion Interpretation

Overstates similarity to other studies without considering local microbiology patterns. Does not explore possible causes for high inappropriate empiric therapy rates. LOS is significant for therapy appropriateness but not MDR status and the reasons are not discussed.

Response

Thank you for your valuable feedback. We have carefully addressed the points raised in our revised manuscript as follows:

1. Local microbiology patterns:

We expanded the discussion to emphasize the influence of regional factors on pathogen distribution and resistance patterns:

"While Staphylococcus aureus remains a leading pathogen globally, the notable presence of Gram-negative bacteria such as Pseudomonas aeruginosa highlights the importance of considering local microbiological patterns that may differ due to regional factors such as environmental conditions and healthcare practices (26)."

2. Causes of high inappropriate empiric therapy rates:

We explored potential reasons for the relatively high rate of inappropriate empirical therapy:

"Reasons for this may include limited access to timely culture and sensitivity results, lack of updated local antibiograms, and variability in clinical decision-making, especially in the absence of standardized local protocols (33)."

3. Length of stay (LOS), therapy appropriateness, and MDR status:

We clarified the relationship between LOS and therapy appropriateness, and discussed why MDR status was not significantly associated with prolonged hospitalization:

"In this study, neither the appropriateness of empirical antibiotic therapy nor the presence of MDR infections showed a statistically significant association with length of hospital stay. This may be explained by the possibility that early modification of antibiotics after culture results reduced the impact of initial inappropriate therapy, and that hospital stay is more strongly influenced by infection severity and other host factors rather than MDR status alone."

Integration with Local Context

Discussion should link observed patterns to local clinical practices (e.g., empiric prescribing habits, lab turnaround times).

Response

We have discussed this by stating:

"Reasons for this may include limited access to timely culture and sensitivity results, lack of updated local antibiograms, and variability in clinical decision-making, especially in the absence of standardized local protocols."

This links observed patterns to local clinical practices such as empiric prescribing habits and lab turnaround times.

Reviewer #2: Overall Recommendation: Major Revisions

Overall Impression:

This manuscript presents a valuable retrospective analysis of diabetic foot infections (DFIs) at a tertiary care center in Jordan. The manuscript is generally well-structured. However, major concerns regarding potential selection bias, data analysis, and the interpretation of results must be addressed before the manuscript can be considered for publication.

Detailed Comments:

1. The exclusion of 79.6% of screened cases (911/1145) of screened cases creates significant selection bias, likely over-representing complex infections. Therefore, the pathogen prevalence and MDR rates cannot be considered representative of the center's true DFI population and must be highlighted as a major limitation.

Response

We thank the reviewer for this important observation. We ag

Attachment Submitted filename: Reply to reviewers.docx

# Peer review: Decision Letter 1

**DOI:** 10.1371/journal.pone.0350837.r003

**Authors:** Shiv Sah

10 Mar 2026

PONE-D-25-38915R1

Multidrug Resistance and Inappropriate Empiric Therapy as Predictors of Hospital Stay in Diabetic Foot Infections

PLOS One

Dear Dr. Itani,

Thank you for submitting your manuscript to PLOS ONE. After careful consideration, we feel that it has merit but does not fully meet PLOS ONE’s publication criteria as it currently stands. Therefore, we invite you to submit a revised version of the manuscript that addresses the points raised during the review process.

Please submit your revised manuscript by Apr 24 2026 11:59PM. If you will need more time than this to complete your revisions, please reply to this message or contact the journal office at plosone@plos.org. When you're ready to submit your revision, log on to https://www.editorialmanager.com/pone/ and select the 'Submissions Needing Revision' folder to locate your manuscript file.

Please include the following items when submitting your revised manuscript:

- A letter that responds to each point raised by the academic editor and reviewer(s). You should upload this letter as a separate file labeled 'Response to Reviewers'.
- A marked-up copy of your manuscript that highlights changes made to the original version. You should upload this as a separate file labeled 'Revised Manuscript with Track Changes'.
- An unmarked version of your revised paper without tracked changes. You should upload this as a separate file labeled 'Manuscript'.

If you would like to make changes to your financial disclosure, please include your updated statement in your cover letter. Guidelines for resubmitting your figure files are available below the reviewer comments at the end of this letter.

If applicable, we recommend that you deposit your laboratory protocols in protocols.io to enhance the reproducibility of your results. Protocols.io assigns your protocol its own identifier (DOI) so that it can be cited independently in the future. For instructions see: https://journals.plos.org/plosone/s/submission-guidelines#loc-laboratory-protocols. Additionally, PLOS ONE offers an option for publishing peer-reviewed Lab Protocol articles, which describe protocols hosted on protocols.io. Read more information on sharing protocols at https://plos.org/protocols?utm_medium=editorial-email&utm_source=authorletters&utm_campaign=protocols.

We look forward to receiving your revised manuscript.

Kind regards,

Shiv Kumar Sah, Master

Academic Editor

PLOS One

**Journal Requirements:**

1. If the reviewer comments include a recommendation to cite specific previously published works, please review and evaluate these publications to determine whether they are relevant and should be cited. There is no requirement to cite these works unless the editor has indicated otherwise.

[Note: HTML markup is below. Please do not edit.]

Reviewers' comments:

Reviewer's Responses to Questions

**Comments to the Author**

1. If the authors have adequately addressed your comments raised in a previous round of review and you feel that this manuscript is now acceptable for publication, you may indicate that here to bypass the “Comments to the Author” section, enter your conflict of interest statement in the “Confidential to Editor” section, and submit your "Accept" recommendation.

Reviewer #3: All comments have been addressed

Reviewer #4: All comments have been addressed

**********

2. Is the manuscript technically sound, and do the data support the conclusions?

The manuscript must describe a technically sound piece of scientific research with data that supports the conclusions. Experiments must have been conducted rigorously, with appropriate controls, replication, and sample sizes. The conclusions must be drawn appropriately based on the data presented.

Reviewer #3: Yes

Reviewer #4: Yes

**********

3. Has the statistical analysis been performed appropriately and rigorously?

Reviewer #3: N/A

Reviewer #4: Yes

**********

4. Have the authors made all data underlying the findings in their manuscript fully available?

The PLOS Data policy requires authors to make all data underlying the findings described in their manuscript fully available without restriction, with rare exception (please refer to the Data Availability Statement in the manuscript PDF file). The data should be provided as part of the manuscript or its supporting information, or deposited to a public repository. For example, in addition to summary statistics, the data points behind means, medians and variance measures should be available. If there are restrictions on publicly sharing data—e.g. participant privacy or use of data from a third party—those must be specified.

Reviewer #3: No

Reviewer #4: No

**********

5. Is the manuscript presented in an intelligible fashion and written in standard English?

PLOS ONE does not copyedit accepted manuscripts, so the language in submitted articles must be clear, correct, and unambiguous. Any typographical or grammatical errors should be corrected at revision, so please note any specific errors here.

Reviewer #3: Yes

Reviewer #4: Yes

**********

6. Review Comments to the Author

Please use the space provided to explain your answers to the questions above. You may also include additional comments for the author, including concerns about dual publication, research ethics, or publication ethics. (Please upload your review as an attachment if it exceeds 20,000 characters)

Reviewer #3: Dear author (s)

I have some revision suggestions below for your manuscript.

The main concern relates to the clarity and consistency of the definitions used in the study—especially the criteria for "appropriate" empiric therapy. The manuscript states that therapy was considered appropriate if at least one antibiotic administered in the first 48 hours covered all isolated pathogens with correct dose, route, and formulation. However, this definition lacks operational detail. It remains unclear how treatment adequacy was judged in polymicrobial infections, how dosing appropriateness was evaluated, and what specific criteria led to classifying 49 cases as “insufficient data.” A more explicit, standardized definition—ideally presented in a table—would greatly enhance reproducibility and transparency.

There is also an issue with the MDR definition. Although the manuscript uses a definition based on resistance to at least one agent in three or more antimicrobial classes, the cited reference does not correspond to the widely accepted Magiorakos et al. (2012, Clinical Microbiology and Infection) framework. This internationally recognized definition should be used and appropriately referenced.

The statistical analysis is descriptive and univariate, relying primarily on Mann–Whitney U tests. Length of stay is influenced by multiple potential confounders, including age, infection severity, comorbidities, presence of osteomyelitis, ICU admission, and number of pathogens isolated. Without a multivariable regression model, the conclusion that inappropriate empiric therapy prolongs hospitalization remains incomplete. A regression approach—linear, Poisson, or negative binomial, depending on LOS distribution—would substantially strengthen the findings.

The flow diagram presented in the manuscript is somewhat confusing. Excluded cases are described narratively but are not shown visually. The diagram would benefit from a clearer PRISMA-style structure detailing screened cases, exclusions with reasons, and final analytic cohorts.

The discussion section provides an overview of relevant literature but would benefit from deeper engagement with the study’s own findings. In particular, the lack of a statistically significant association between MDR infections and length of stay warrants a more nuanced explanation. The sustained use of broad-spectrum therapy despite availability of culture results is important and could be explored more thoroughly in terms of stewardship implications, local prescribing culture, and institutional factors.

Several tables and figures require clearer formatting, more descriptive legends, and more precise labeling. Some variables in Tables 1 and 2 are presented in ways that may confuse readers, and percentages sometimes do not sum intuitively. Presenting the transitions between empiric and definitive therapy in a table (rather than solely in the text) would also improve clarity.

Minor issues include occasional repetition, slight inconsistencies in terminology, and referencing concerns. The manuscript is generally well written, but some sections—especially the introduction and discussion—could be more concise. The abstract could be shortened for clarity. Microorganism names should be written in italic. It should be corrected in all sections.

Ethical considerations are adequately addressed, although the data availability statement may require refinement to fully comply with PLOS ONE expectations. It may help to clarify why anonymization alone is insufficient to meet public data-sharing requirements.

In its current form, the manuscript contributes valuable descriptive epidemiology but requires substantial methodological refinement and clarification. With careful revision, including clearer definitions, improved statistical modeling, more structured data presentation, and deeper interpretation, the study would be suitable for reconsideration.

Reviewer #4: The author(s) have adequately addressed the comments raised in the previous review. However, the following needs to be addressed as well:

RESULTS

Table 1:

Comment:

•Ensure open and closed brackets around the percentages under the Frequency column

•Correct the proportions/percentages of the following categories:

Most common illnesses – Correct the proportion for Chronic kidney failure – it is not 26%.

•Was the patient transferred to ICU – Correct the proportion of ‘No’ – it is not 84.3%

3.4 Isolated Bacteria.

Correct the percentage of Enteroccocus spp - 51(51.8%) to the nearest decimal point.

3.5 3.5. Empirical and Definitive Antimicrobial Therapy

Correct the percentage of ‘Most antibiotics were administered intravenously (n-179; 76.5%) to the nearest decimal point.

Line 265: You state ‘Following culture and susceptibility results, antibiotics were modified in several ways’.

Comment: This text implies that antibiotics were modified, which is not the case. I suppose what was modified was the antibiotic therapy.

Fig 2 – The flow of data is confusing after the 3rd level category. The implication is that the numbers in the box below a category are a subset of that category, which is not the case.

Line 300: You state ‘#using simple linear regression’. For clarity, I suggest you state ‘Using univariable linear regression’ to mean you regressed each variable separately.

Line 305: You state ‘This study explored the epidemiology, causative pathogens, resistance patterns, and treatment 306 approaches for DFI at a tertiary care center in Jordan.’.

Comment: Epidemiology is a quite broad concept and appears here in the discussion without any reference to it in the title or aim of your study.

Line 306-308: You state ‘A total of 234 patients participated, with 307 males representing 75.6% (n=177) and females 24.4% (n=57), emphasizing sex differences in DFI incidence and management (20)’.

Comment: A difference should be supported with a p-value showing significance.

Line 366: You state ‘The sample size limited statistical power for subgroup analyses, especially for MDR infections

Comment: It would be desirable to show the post-hoc power of this study based on the sample size used. This comment was also raised previously.

Line 378: Spelling of Klebsiella pneumoniae should be corrected

**********

7. PLOS authors have the option to publish the peer review history of their article (what does this mean?). If published, this will include your full peer review and any attached files.

If you choose “no”, your identity will remain anonymous but your review may still be made public.

**Do you want your identity to be public for this peer review?** For information about this choice, including consent withdrawal, please see our Privacy Policy.

Reviewer #3: No

Reviewer #4: **Yes:** Professor George Nasinyama

**********

[NOTE: If reviewer comments were submitted as an attachment file, they will be attached to this email and accessible via the submission site. Please log into your account, locate the manuscript record, and check for the action link "View Attachments". If this link does not appear, there are no attachment files.]

To ensure your figures meet our technical requirements, please review our figure guidelines: https://journals.plos.org/plosone/s/figures

You may also use PLOS’s free figure tool, NAAS, to help you prepare publication quality figures: https://journals.plos.org/plosone/s/figures#loc-tools-for-figure-preparation.

NAAS will assess whether your figures meet our technical requirements by comparing each figure against our figure specifications.

Attachment Submitted filename: PLOS one my reviewer comments.docx

# Author comment: Author response to Decision Letter 2

**DOI:** 10.1371/journal.pone.0350837.r004

18 Apr 2026

Review Comments to the Author

Reviewer #3: Dear author (s)

I have some revision suggestions below for your manuscript.

The main concern relates to the clarity and consistency of the definitions used in the study—especially the criteria for "appropriate" empiric therapy. The manuscript states that therapy was considered appropriate if at least one antibiotic administered in the first 48 hours covered all isolated pathogens with correct dose, route, and formulation. However, this definition lacks operational detail. It remains unclear how treatment adequacy was judged in polymicrobial infections, how dosing appropriateness was evaluated, and what specific criteria led to classifying 49 cases as “insufficient data.” A more explicit, standardized definition—ideally presented in a table—would greatly enhance reproducibility and transparency.

Response

We agree with the reviewer’s assessment and have revised Section 2.4 to provide a standardized operational definition based on contemporary literature (Van Heuverswyn et al., 2023). Specifically, we adopted a 'strict-concordance' model where appropriateness required 100% coverage of all isolates in polymicrobial infections, correct renal dosing, and administration within 48 hours. As suggested, we have added Table 1 to clearly outline the decision matrix used for these classifications and to detail the exclusion criteria for the 49 'insufficient data' cases."

Table 1. Operational Definitions for Empirical Antibiotic Appropriateness

Category Criteria for "Appropriate" Classification

Microbiological Concordance All isolated pathogens must be susceptible in vitro to at least one empirical agent. In polymicrobial infections, 100% of isolates must be covered (15).

Dosing & Route Dose, formulation, and route must align with IWGDF/IDSA 2024 standards, adjusted for admission renal function (12).

Timing First dose must be administered within 48 hours of hospital admission.

Insufficient Data Assigned when susceptibility results were incomplete for any isolate or when medication administration timestamps were missing from the electronic record.

There is also an issue with the MDR definition. Although the manuscript uses a definition based on resistance to at least one agent in three or more antimicrobial classes, the cited reference does not correspond to the widely accepted Magiorakos et al. (2012, Clinical Microbiology and Infection) framework. This internationally recognized definition should be used and appropriately referenced.

Response

We thank the reviewer for this important comment. We have revised the manuscript to adopt the internationally accepted definition of multidrug resistance as proposed by Magiorakos et al. (2012, Clinical Microbiology and Infection), and the previous reference has been replaced accordingly.

The statistical analysis is descriptive and univariate, relying primarily on Mann–Whitney U tests. Length of stay is influenced by multiple potential confounders, including age, infection severity, comorbidities, presence of osteomyelitis, ICU admission, and number of pathogens isolated. Without a multivariable regression model, the conclusion that inappropriate empiric therapy prolongs hospitalization remains incomplete. A regression approach—linear, Poisson, or negative binomial, depending on LOS distribution—would substantially strengthen the findings.

Response

We agree that length of stay (LOS) is influenced by multiple clinical factors, and we have addressed this by conducting a multivariate linear regression analysis using log-transformed LOS as the dependent variable to account for skewness.

To adjust for confounding, we included a comprehensive set of clinically relevant variables in the model. These included age, gender, smoking status, type of diabetes, HbA1c levels, department of admission (internal medicine vs surgery department), ICU transfer during hospitalization, presence of diabetic foot ulcers, infection severity based on the IWGDF/IDSA classification, appropriateness of empiric antibiotic therapy, and the presence of MDR infection.

The Results section was updated to include Table 3, and the corresponding text was revised as follows:

“A multivariate linear regression analysis was conducted to identify factors associated with the log-transformed length of hospital stay among patients with DFIs (Table 3). In the univariate analysis, seven predictors had P values less than 0.250, making them eligible for inclusion in the multivariate linear regression model. These included type of diabetes (P = 0.105), department of admission (P = 0.048), ICU transfer (P = 0.011), presence of diabetic foot ulcer (P = 0.051), infection severity based on the IWGDF/IDSA classification (P = 0.015), appropriate empiric therapy (P = 0.234), and presence of MDR infection (P = 0.069).

After adjusting for covariates in the multivariate model, only the severity of infection remained a statistically significant predictor. Patients with less severe infections (IWGDF/IDSA Grader 2) had significantly shorter hospital stays compared to those with more severe infections (Grade 3 or 4; β = -0.161, P = 0.034).”

The flow diagram presented in the manuscript is somewhat confusing. Excluded cases are described narratively but are not shown visually. The diagram would benefit from a clearer PRISMA-style structure detailing screened cases, exclusions with reasons, and final analytic cohorts.

Response

We thank the reviewer for this constructive suggestion. We agree that a visual representation of the screening and exclusion process enhances the clarity of the study’s methodology.

In response, we have revised Figure 1 to follow a PRISMA-style format. The updated figure now explicitly illustrates the flow from the initial identification of cases (n=1,145) to the final analytic cohort (n=234). Specifically, we have added an exclusion box that details the 911 cases excluded, categorized by reason (lack of adequate data, negative culture results, outpatient management, and concurrent infections). We have also streamlined the visual elements and added phase labels (Identification, Screening and Exclusions, and Analysis) to meet standard journal reporting requirements. We believe this revision provides a more transparent and comprehensive overview of our study population.

The discussion section provides an overview of relevant literature but would benefit from deeper engagement with the study’s own findings. In particular, the lack of a statistically significant association between MDR infections and length of stay warrants a more nuanced explanation. The sustained use of broad-spectrum therapy despite availability of culture results is important and could be explored more thoroughly in terms of stewardship implications, local prescribing culture, and institutional factors.

Response

We thank the reviewer for this constructive critique. The Discussion section has been extensively revised to provide a deeper, more nuanced engagement with our primary findings. Specifically, we have added an analysis of the 'neutralization effect,' where aggressive early empirical therapy may have mitigated the expected impact of MDR pathogens on hospitalization duration. Furthermore, we have introduced a critical exploration of 'clinical inertia' and institutional stewardship barriers—such as the lack of formalized multidisciplinary 'antibiotic timeouts'—to explain the sustained use of broad-spectrum therapy despite culture availability

Several tables and figures require clearer formatting, more descriptive legends, and more precise labeling. Some variables in Tables 1 and 2 are presented in ways that may confuse readers, and percentages sometimes do not sum intuitively. Presenting the transitions between empiric and definitive therapy in a table (rather than solely in the text) would also improve clarity.

Response

All tables and figures have been revised for improved clarity and precision. Variable labeling and legends were updated to ensure technical accuracy, and all percentages were audited and corrected to ensure intuitive summation. As suggested, a new table (Table 4) has been added to clearly present the transitions from empirical to definitive therapy

Minor issues include occasional repetition, slight inconsistencies in terminology, and referencing concerns. The manuscript is generally well written, but some sections—especially the introduction and discussion—could be more concise.

Response

We thank the reviewer for this comment. The manuscript has been carefully revised to reduce repetition, ensure consistency in terminology, and address referencing concerns. The Introduction and Discussion sections have also been refined for improved clarity and conciseness.

The abstract could be shortened for clarity.

Response

The abstract has been shortened for improved clarity and focus.

Microorganism names should be written in italic. It should be corrected in all sections.

Response

All microorganism names have been revised and formatted in italics throughout the manuscript.

Ethical considerations are adequately addressed, although the data availability statement may require refinement to fully comply with PLOS ONE expectations.

Response

We thank the reviewer for this comment. The Data Availability Statement has been revised to fully comply with PLOS ONE requirements by clearly specifying the restrictions on data sharing and the process for accessing the data through the Institutional Review Board of Jordan University Hospital.

It may help to clarify why anonymization alone is insufficient to meet public data-sharing requirements.

Response

We thank the reviewer for this valuable suggestion. We have clarified that anonymization alone is insufficient in this study due to the inclusion of detailed clinical and hospitalization data, which may carry a risk of indirect patient re-identification. Therefore, access to the dataset is restricted and subject to Institutional Review Board approval to ensure compliance with ethical and confidentiality standards.

In its current form, the manuscript contributes valuable descriptive epidemiology but requires substantial methodological refinement and clarification. With careful revision, including clearer definitions, improved statistical modeling, more structured data presentation, and deeper interpretation, the study would be suitable for reconsideration.

Response

We thank the reviewer for identifying areas for improvement. We have revised the manuscript to ensure high methodological rigor. Specifically, we:

• Operationalized Definitions: Adopted a strict 'all-or-nothing' concordance model for antibiotic appropriateness (Van Heuverswyn et al., 2023) and added Table 1 for transparency.

• Strengthened Statistics: Implemented log-transformation for Length of Stay (LOS) in our regression models to correct for data skewness.

• Clarified Exclusions: Defined specific criteria for the 49 'insufficient data' cases to prevent selection bias.

• Enhanced Structure: Reorganized data presentation to ensure all clinical and microbiological outcomes are logically linked to our study objectives.

Reviewer #4: The author(s) have adequately addressed the comments raised in the previous review. However, the following needs to be addressed as well:

RESULTS

Table 1:

Comment:

•Ensure open and closed brackets around the percentages under the Frequency column

Response

All percentages under the Frequency column have been checked, and the use of open and closed brackets has been corrected throughout the manuscript.

•Correct the proportions/percentages of the following categories:

Most common illnesses – Correct the proportion for Chronic kidney failure – it is not 26%.

Response

The proportion for chronic kidney failure under the “Most common illnesses” category has been reviewed and corrected to 22.2% in the revised manuscript.

•Was the patient transferred to ICU – Correct the proportion of ‘No’ – it is not 84.3%

Response

The proportion of patients who were not transferred to the ICU has been reviewed and corrected to 84.6% in the revised manuscript.

3.4 Isolated Bacteria.

Correct the percentage of Enteroccocus spp - 51(51.8%) to the nearest decimal point.

Response

We thank the reviewer for this comment. The percentage of Enterococcus spp. has been reviewed and corrected to 21.8% in the revised manuscript.

3.5 Empirical and Definitive Antimicrobial Therapy

Correct the percentage of ‘Most antibiotics were administered intravenously (n-179; 76.5%) to the nearest decimal point.

Response

The percentage for “Most antibiotics were administered intravenously” has been reviewed and confirmed to be correct at 76.5% in the revised manuscript.

Line 265: You state ‘Following culture and susceptibility results, antibiotics were modified in several ways’. Comment: This text implies that antibiotics were modified, which is not the case. I suppose what was modified was the antibiotic therapy.

Response

We thank the reviewer for this comment. The text has been revised to clarify that “antibiotic therapy,” rather than “antibiotics,” was modified following culture and susceptibility results.

Fig 2 – The flow of data is confusing after the 3rd level category. The implication is that the numbers in the box below a category are a subset of that category, which is not the case.

Response

We appreciate the reviewer’s feedback regarding the visual clarity of the data flow. We have redesigned Figure 2 to follow a standard PRISMA-style branching logic. The vertical 'stacking' has been replaced with horizontal branching to explicitly show that categories (Appropriate, Inappropriate, and Inadequate data) are mutually exclusive subsets of the same parent cohort.

Line 300: You state ‘#using simple linear regression’. For clarity, I suggest you state ‘Using univariable linear regression’ to mean you regressed each variable separately.

Response

We thank the reviewer for this comment. The text has been revised to “using univariate linear regression” to clarify that each variable was analyzed separately.

Line 305: You state ‘This study explored the epidemiology, causative pathogens, resistance patterns, and treatment approaches for DFI at a tertiary care center in Jordan.’

Comment: Epidemiology is a quite broad concept and appears here in the discussion without any reference to it in the title or aim of your study.

Response

We thank the reviewer for this comment. We have revised the sentence to avoid the use of the term “epidemiology,” as it was not explicitly defined as an aim of the study. The text has been modified accordingly to better align with the study objectives as follows:

“This study explored the causative pathogens, antimicrobial resistance patterns, and treatment approaches for DFIs at a tertiary care center in Jordan.”

Line 306-308: You state ‘A total of 234 patients participated, with 307 males representing 75.6% (n=177) and females 24.4% (n=57), emphasizing sex differences in DFI incidence and management (20)’. Comment: A difference should be supported with a p-value showing significance.

Response

We thank the reviewer for this comment. We have revised the sentence by removing the interpretation of sex differences, as no statistical comparison was performed to assess significance between males and females in the study.

Line 366: You state ‘The sample size limited statistical power for subgroup analyses, especially for MDR infections. Comment: It would be desirable to show the post-hoc power of this study based on the sample size used. This comment was also raised previously.

Response

We thank the reviewer for this important comment. Based on the final multivariate regression model (R² = 0.071, f² = 0.076), a post-hoc power analysis indicated that the study achieved an overall statistical power of approximately 0.88 at an alpha level of 0.05. We acknowledge that, despite adequate overall power, subgroup analyses—particularly for MDR infections—may still be underpowered due to limited sample size.

The limitation was revised as follows:

“A post hoc analysis shows the overall regression model has limited explanatory power (R2 = 0.071) but still had acceptable overall statistica

Attachment Submitted filename: Reply_to_Reviewers_auresp_2.docx

# Editor report: Decision Letter 2

**DOI:** 10.1371/journal.pone.0350837.r005

**Authors:** Shiv Sah

19 May 2026

Multidrug Resistance and Inappropriate Empiric Therapy as Predictors of Hospital Stay in Diabetic Foot Infections

PONE-D-25-38915R2

Dear Dr. Itani,

We’re pleased to inform you that your manuscript has been judged scientifically suitable for publication and will be formally accepted for publication once it meets all outstanding technical requirements.

Within one week, you’ll receive an e-mail detailing the required amendments. When these have been addressed, you’ll receive a formal acceptance letter and your manuscript will be scheduled for publication.

An invoice will be generated when your article is formally accepted. Please note, if your institution has a publishing partnership with PLOS and your article meets the relevant criteria, all or part of your publication costs will be covered. Please make sure your user information is up-to-date by logging into Editorial Manager at Editorial Manager® and clicking the ‘Update My Information' link at the top of the page. For questions related to billing, please contact billing support.

If your institution or institutions have a press office, please notify them about your upcoming paper to help maximize its impact. If they’ll be preparing press materials, please inform our press team as soon as possible -- no later than 48 hours after receiving the formal acceptance. Your manuscript will remain under strict press embargo until 2 pm Eastern Time on the date of publication. For more information, please contact onepress@plos.org.

Kind regards,

Shiv Kumar Sah, Master

Academic Editor

PLOS One

Additional Editor Comments (optional):

Reviewers' comments:

# Editor report: Acceptance letter

**DOI:** 10.1371/journal.pone.0350837.r006

**Authors:** Shiv Sah

PONE-D-25-38915R2

PLOS One

Dear Dr. Itani,

I'm pleased to inform you that your manuscript has been deemed suitable for publication in PLOS One. Congratulations! Your manuscript is now being handed over to our production team.

At this stage, our production department will prepare your paper for publication. This includes ensuring the following:

* All references, tables, and figures are properly cited

* All relevant supporting information is included in the manuscript submission,

* There are no issues that prevent the paper from being properly typeset

You will receive further instructions from the production team, including instructions on how to review your proof when it is ready. Please keep in mind that we are working through a large volume of accepted articles, so please give us a few days to review your paper and let you know the next and final steps.

Lastly, if your institution or institutions have a press office, please let them know about your upcoming paper now to help maximize its impact. If they'll be preparing press materials, please inform our press team within the next 48 hours. Your manuscript will remain under strict press embargo until 2 pm Eastern Time on the date of publication. For more information, please contact onepress@plos.org.

You will receive an invoice from PLOS for your publication fee after your manuscript has reached the completed accept phase. If you receive an email requesting payment before acceptance or for any other service, this may be a phishing scheme. Learn how to identify phishing emails and protect your accounts at https://explore.plos.org/phishing.

If we can help with anything else, please email us at customercare@plos.org.

Thank you for submitting your work to PLOS ONE and supporting open access.

Kind regards,

PLOS ONE Editorial Office Staff

on behalf of

Mr Shiv Kumar Sah

Academic Editor

PLOS One
